# Functional identification of bacterial spermine, thermospermine, norspermine, norspermidine, spermidine, and *N*^1^-aminopropylagmatine synthases

**DOI:** 10.1016/j.jbc.2024.107281

**Published:** 2024-04-06

**Authors:** Bin Li, Jue Liang, Hamid R. Baniasadi, Shin Kurihara, Margaret A. Phillips, Anthony J. Michael

**Affiliations:** 1 Department of Biochemistry, UT Southwestern Medical Center, Dallas, Texas, USA; 2 Faculty of Biology-Oriented Science and Technology, Kindai University, Kinokawa, Wakayama, Japan

**Keywords:** bacterial metabolism, biosynthesis, polyamine, spermidine, spermine, thermospermine, *N*^1^-aminopropylagmatine, norspermidine, norspermine

## Abstract

Spermine synthase is an aminopropyltransferase that adds an aminopropyl group to the essential polyamine spermidine to form tetraamine spermine, needed for normal human neural development, plant salt and drought resistance, and yeast CoA biosynthesis. We functionally identify for the first time bacterial spermine synthases, derived from phyla Bacillota, Rhodothermota, Thermodesulfobacteriota, Nitrospirota, Deinococcota, and Pseudomonadota. We also identify bacterial aminopropyltransferases that synthesize the spermine same mass isomer thermospermine, from phyla Cyanobacteriota, Thermodesulfobacteriota, Nitrospirota, Dictyoglomota, Armatimonadota, and Pseudomonadota, including the human opportunistic pathogen *Pseudomonas aeruginosa*. Most of these bacterial synthases were capable of synthesizing spermine or thermospermine from the diamine putrescine and so possess also spermidine synthase activity. We found that most thermospermine synthases could synthesize tetraamine norspermine from triamine norspermidine, that is, they are potential norspermine synthases. This finding could explain the enigmatic source of norspermine in bacteria. Some of the thermospermine synthases could synthesize norspermidine from diamine 1,3-diaminopropane, demonstrating that they are potential norspermidine synthases. Of 18 bacterial spermidine synthases identified, 17 were able to aminopropylate agmatine to form *N*^1^-aminopropylagmatine, including the spermidine synthase of *Bacillus subtilis*, a species known to be devoid of putrescine. This suggests that the *N*^1^-aminopropylagmatine pathway for spermidine biosynthesis, which bypasses putrescine, may be far more widespread than realized and may be the default pathway for spermidine biosynthesis in species encoding L-arginine decarboxylase for agmatine production. Some thermospermine synthases were able to aminopropylate *N*^1^-aminopropylagmatine to form *N*^12^-guanidinothermospermine. Our study reveals an unsuspected diversification of bacterial polyamine biosynthesis and suggests a more prominent role for agmatine.

Polyamines are amino acid–derived, small polycations synthesized by bacteria, archaea, eukaryotes, and some viruses (1, 2, 3). Most linear polyamines are synthesized from the diamine putrescine (Put) by sequential transfer of one or more aminopropyl groups (Fig. 1) mediated by a small family of aminopropyltransferases (APTs) that have a common evolutionary origin, including spermidine (Spd), spermine (Spm), and thermospermine (Tspm) synthases (4, 5, 6, 7, 8). The aminopropyl groups are donated by decarboxylated *S*-adenosylmethionine (dcAdoMet) (9), formed by *S*-adenosylmethionine decarboxylase (AdoMetDC) (Fig. 1*A*). It is likely that Spd synthase (SpdSyn) was present in the Last Universal Common Ancestor of all life (10), indicating the primordial role of Spd in early cellular physiology. In eukaryotes and archaea, Spd is essential for cell growth and proliferation due to its role in the deoxyhypusine/hypusine posttranslational modification of translation factor eIF5a/aIF5a (11, 12, 13). The essentiality of Spd in bacterial growth varies between species (14), likely reflecting the diversification of Spd function across evolutionary time.

**Figure 1:**
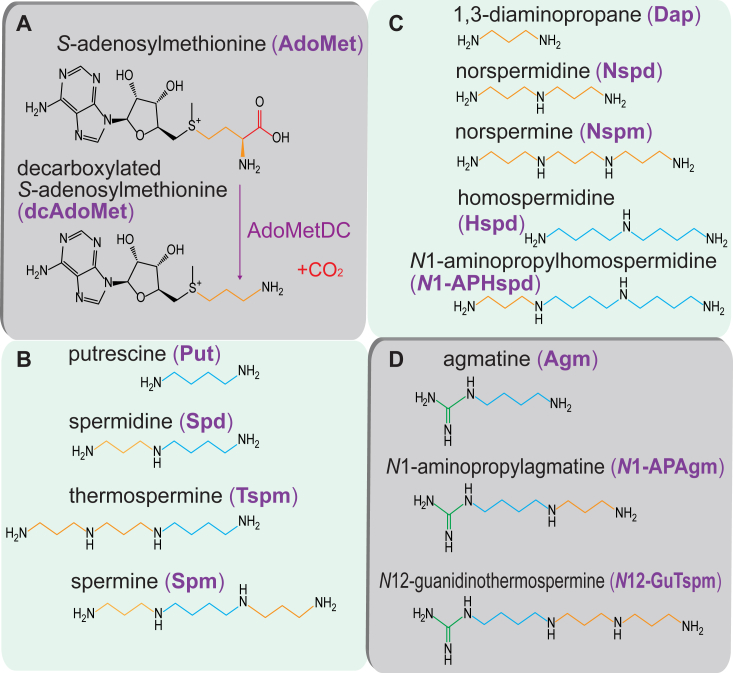
**Aminopropyltransferase substrates and products.***A*, production of decarboxylated *S*-adenosylmethionine (dcAdoMet) from *S*-adenosylmethionine (AdoMet) by *S*-adenosylmethionine decarboxylase (AdoMetDC). The donated aminopropyl group is shown in *tan*. *B*, consecutive aminopropylations produce spermidine (Spd) from putrescine (Put, *blue*) and spermine (Spm) and thermospermine (Tspm) from Spd. *C*, consecutive aminopropylations produce norspermidine (Nspd) from 1,3-diaminopropane (Dap), norspermine (Nspm) from Nspd, and *N*^1^-aminopropylhomospermidine (*N*^1^-APHspd) from homospermidine (Hspd). *D*, consecutive aminopropylations produce *N*^1^-aminopropylagmatine (*N*^1^-APAgm) from agmatine (Agm) and *N*^12^-guanidinothermospermine (*N*^12^-GuTspm) from *N*^1^-APAgm. The guanidino group is shown in *green*.

In eukaryotes, in addition to the triamine Spd, the tetraamine Spm (Fig. 1*B*) has evolved from Spd by aminopropylation of the *N*^8^-aminobutyl side of Spd, performed by Spm synthase (SpmSyn). Spm biosynthesis has evolved independently at least three times in eukaryotes: in metazoa, fungi, and plants (15, 16). The function of Spm is less understood than that of Spd; it is not universally present in all eukaryotes but it is known to regulate potassium channels and NMDA receptors (17). Mutations of the human SpmSyn result in severe mental developmental defects (18), although it is not clear whether the problem is due to Spm deficiency or Spd excess (19). In the model flowering plant *Arabidopsis thaliana*, Spm is not required for normal growth but mutants of SpmSyn are highly sensitive to drought and salt stress (20, 21). In the yeast *Saccharomyces cerevisiae*, SpmSyn is not required for growth unless the growth medium lacks pantothenate (22). Spm is oxidized by yeast FMS1 to produce 1,3-diaminopropane (Fig. 1*C*), which is converted to β-alanine, pantothenate, and then co-enzyme A (23), revealing that Spm is required for *de novo* CoA biosynthesis.

The fact that Spm biosynthesis has evolved independently at least three times in eukaryotes suggests selective pressures to evolve Spm biosynthesis for diverse cellular processes. In addition to Spm, eukaryotes of the algal and plant lineage produce a structural isomer of Spm with the same mass—Tspm, which is synthesized from Spd by addition of an aminopropyl group to the *N*^1^-aminopropyl side of Spd (Fig. 1*B*) (24). A mutant of TspmSyn (*acl5*) in *A. thaliana*, which was originally misidentified as a SpmSyn (25, 26), exhibits severe growth abnormalities, dwarfism, and aberrant vascular development. The structural analog norspermine (Fig. 1*C*) is able to partially replace Tspm function in growth and development (27). Tspm has been shown recently to be essential for correct organ development in the nonvascular plant *Marchantia polymorpha* (28).

It has been proposed that bacteria do not encode SpmSyn (15), but many phylogenetically diverse bacteria, especially thermophiles, have been found to contain Spm (29, 30, 31, 32). The APT of hyperthermophile *Thermus thermophilus* exhibits a very relaxed substrate specificity and produces a large number of longer polyamines by aminopropylation of diverse polyamine substrates (33, 34). However, no specific SpmSyn has been identified in bacteria. Furthermore, previous measurements of Spm in bacteria did not usually distinguish between Spm and Tspm due to their similar chromatographic behavior. Similarly, no specific TspmSyn has been identified in bacteria, although the APT of *Thermus thermophilus* is regarded as a bifunctional *N*^1^-aminopropylagmatine/Tspm synthase (7, 33). Given the known presence of Spm and Tspm in bacteria, we sought to determine whether bacteria encode functional Spm and Tspm synthases.

## Results

### Selecting candidates for bacterial spermine and thermospermine synthases

Bacteria that accumulate Spm/Tspm have been identified in a long-term effort to use polyamine profiles for chemotaxonomy (29, 30, 31, 32). We selected diverse bacterial species that were found by Hamana *et al.* to accumulate Spm/Tspm and interrogated their genomes for APT-encoding genes. Most species we selected encode pairs of APT homologs, but some encode single (“singleton”) APT proteins (Table S1). As *Escherichia coli* does not produce Spm/Tspm, it provides a useful platform for detection of SpmSyn/TspmSyn activity. The selected genes were synthesized for the expression in *E. coli* BL21 from plasmid pETDuet-1. As positive controls for Spm, Tspm, and Spd production, the biochemically validated eukaryotic SpmSyns of *Homo sapiens* (35) and flowering plant *A. thaliana* (36), TspmSyns of *A. thaliana* (25, 26) and chlorophyte single-celled alga *Chlamydomonas reinhardtii* (37), and SpdSyns of *H. sapiens*, *A. thaliana*, and corresponding homolog from *C. reinhardtii* were synthesized (Table S1) and expressed in *E. coli* BL21-derived strains.

Some of the bacterial genes were encoded by species from the Bacillota phylum: *Thermanaerobacter brockii* (1 APT, thermophile (T)), *Thermosyntropha lipolytica* (2 APTs, T), *Thermobrachium celere* (1 APT, T), *Sulfobacillus acidophilus* (2 APTs, T), *Geobacillus stearothermophilus* (2 APTs, T), and *Desulfosporosinus orientis* (2 APTs, mesophile (M)). Others were encoded by phyla Cyanobacteriota, *Arthrospira platensis* (Spirulina, 2 APTs, M); Thermodesulfobacteriota, *Thermodesulfobacterium thermophilum* (2 APTs, T); Thermotogota, *Thermotoga maritima* (1 APT, T); Deinococcota, *Oceanithermus profundus* (1 APT, T) and *Thermus thermophilus* (1 APT, T); Nitrospirota, *Thermodesulfovibrio yellowstonii* (2 APTs, T); Rhodothermota, *Rhodothermus marinus* (2 APTs, T); and Dictyoglomota, *Dictyoglomus thermophilum* (2 APTs, T). We chose a singleton APT encoded by the genome of cyanobacterium *Microcystis aeruginosa* (M), a species that was found not to accumulate Spm/Tspm (32) but the APT is closely homologous to the two APTs encoded by *A. platensis*. In addition to species investigated by Hamana *et al.*, we also selected a number of mesophilic species among many based on the presence of two APT homologs in a genome: *Desulfoarculus baarsii* (Thermodesulfobacteriota), *Fimbriimonas ginsengsoli* (Armatimonadota), *Heliorestis convoluta* (Bacillota), and *Pseudomonas aeruginosa* PAO1 (Pseudomonadota). We also selected three mesophilic bacterial species encoding single divergent APTs: *Leptospirillum ferrodiazotrophum* (Nitrospirota); and *Candidatus Pelagibacter ubique* HTCC1062 and *Ca. Pelagibacter* sp. HTCC7211 (Pseudomonadota) that each encode a fused N-terminal AdoMetDC (class 1b) (38) and C-terminal APT.

### Functional identification of bacterial spermine and thermospermine synthases

**Figure 2:**
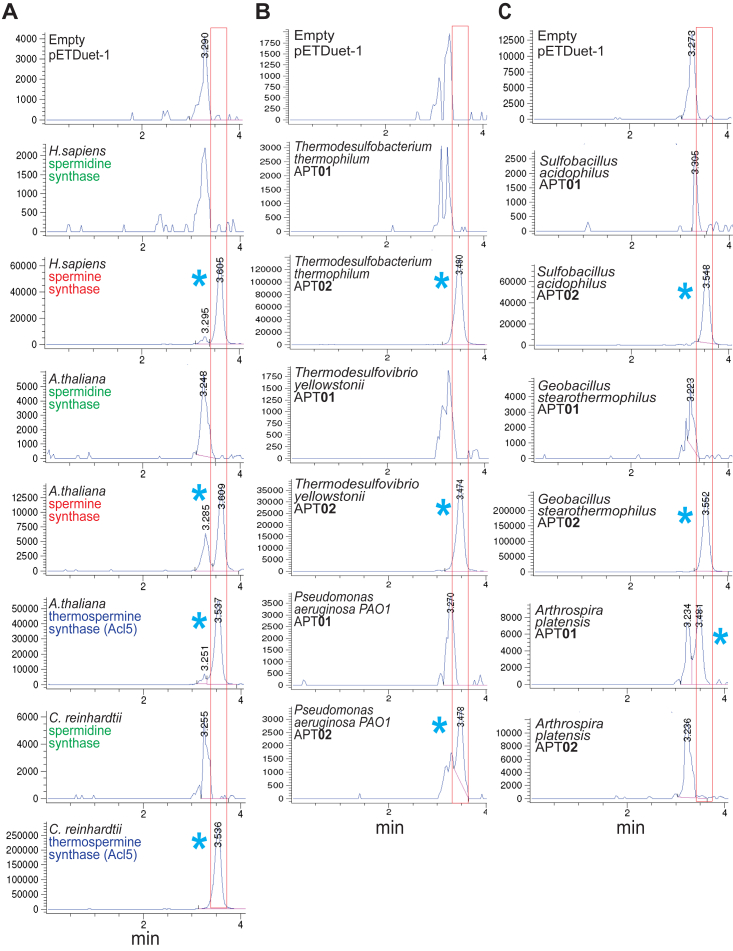
**Expression of aminopropyltransferases in *Escherichia coli* BL21*speG*.***A*–*C*, samples in each vertical panel represent independent experiments (group *A*, group *B*, group *C*). APT01 and APT02 represent individual genes from pairs of aminopropyltransferase homologs encoded in the same genome. Shown are the Extracted Ion Chromatograms for tetrabenzoylated Spm/Tspm (EIC = 619.02:620.02). The *red* box outlines the position of eluted tetrabenzoylated Spm/Tspm detected by LC-MS. The *blue* asterisk indicates the presence of peaks for tetrabenzoylated Spm/Tspm (m/z 619.6). *E. coli* BL21*speG* synthesizes spermidine. Aminopropyltransferase genes were expressed from pETDuet-1. The vertical axis represents arbitrary units of ion intensity. APT, aminopropyltransferase.

**Figure 3:**
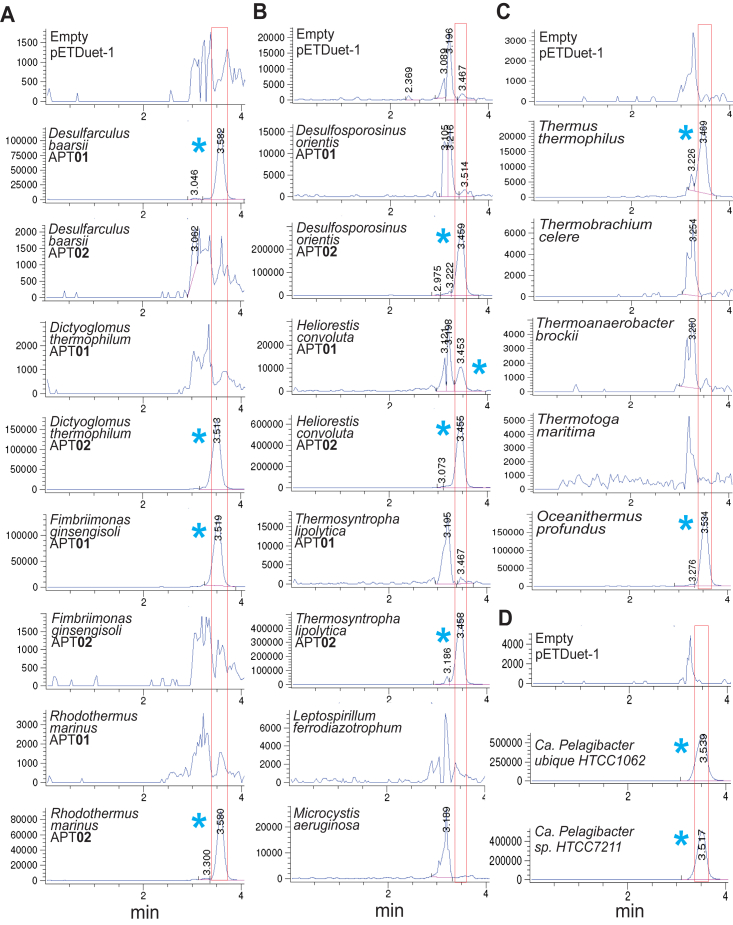
**Expression of aminopropyltransferases in *Escherichia coli* BL21*speG*.***A*–*D*, samples in each vertical panel represent independent experiments (group *A*, group *B*, group *C*, group *D*). APT01 and APT02 represent individual genes from pairs of aminopropyltransferase homologs encoded in the same genome; others represent singleton aminopropyltransferases. See Figure 2 for further description. APT, aminopropyltransferase.

Spm is much more efficiently *N*-acetylated by bacterial Spd *N*-acetyltransferase (SpeG) than is Spd (39). We therefore expressed each APT gene in a Δ*speG* deletion strain of *E. coli* BL21 to avoid *N*-acetylation of any Spm or Tspm that might be produced. After growth in polyamine-free, chemically-defined M9 medium and induced expression of each gene, polyamines were extracted from cells and benzoylated for analysis by LC-MS. The relative efficiency of each APT enzyme for producing Spm/Tspm cannot be directly compared using our approach due to possible differences in steady-state expression level of each APT gene expressed from pETDuet-1. The control BL21*speG* strain expressing the empty pETDuet-1 plasmid contains Put and Spd, and we looked for the production of tetrabenzoylated Spm/Tspm (extracted ion chromatogram (EIC) mass tolerance window 619:620 Da). In this LC-MS system, the Spm/Tspm identical mass structural isomers are not clearly distinguished chromatographically. Eukaryotic *H. sapiens* and *A. thaliana* SpmSyns and *A. thaliana* and *C. reinhardtii* Tspmsyns produced Spm/Tspm when expressed in BL21*speG* (Fig. 2), validating the utility of *E. coli* for detecting Spm/Tspm synthase-encoding genes (Table 1). The *A. thaliana*, *C. reinhardtii*, and *H. sapiens* SpdSyns did not produce detectable Spm/Tspm. Within the pairs of bacterial APT homologs analyzed, in each case, one gene produced a peak corresponding to Spm/Tspm (Figs. 2 and 3, Table 1). Among the singleton APTs, those encoded by *Thermus thermophilus*, *Oceanithermus* *profundus*, and the two *Ca. Pelagibacter* species produced Spm/Tspm (Fig. 3).

**Table 1:** Spermidine and spermine/thermospermine synthase activities of aminopropyltransferase homologs

Species (Phylum)	Protein [Genbank acc.no.]	SpdSyn	Spm/TspmSyn
*Arthrospira platensis* (Cyanobacteriota)	APT01 [BAI92260]	Y	Y
*Arthrospira platensis* (Cyanobacteriota)	APT02 [BAI90257]	Y	N
*Desulfarculus baarsii* (Thermodesulfobacteriota)	APT01 [WP_013259899]	Y	Y
*Desulfarculus baarsii* (Thermodesulfobacteriota)	APT02 [WP_013257411]	Y	N
*Desulfosporosinus orientis* (Bacillota, Clostridia)	APT01 [WP_014182696]	Y	N
*Desulfosporosinus orientis* (Bacillota, Clostridia)	APT02 [WP_014182917]	Y	Y
*Dictyoglomus thermophilum* (Dictyoglomota)	APT01 [WP_012546901]	Y	N
*Dictyoglomus thermophilum* (Dictyoglomota)	APT02 [WP_012547113]	Y	Y
*Fimbriimonas ginsengisoli* (Armatimonadota)	APT01 [WP_025227485]	Y	Y
*Fimbriimonas ginsengisoli* (Armatimonadota)	APT02 [WP_025225227]	Y	N
*Geobacillus stearothermophilus* (Bacillota, Bacilli)	APT01 [WP_049624640]	Y	N
*Geobacillus stearothermophilus* (Bacillota, Bacilli)	APT02 [WP_033014118]	Y	Y
*Heliorestis convoluta* (Bacillota, Clostridia)	APT01 [WP_153726121]	Y	T
*Heliorestis convoluta* (Bacillota, Clostridia)	APT02 [WP_153723875]	Y	Y
*Leptospirillum ferrodiazotrophum* (Nitrospirota)	APT [EES53973]	Y	∗
*Microcystis aeruginosa* (Cyanobacteriota)	APT [WP_002790803]	Y	N
*Oceanithermus profundus* (Deinococcota)	APT [WP_013456826]	Y	Y
*Ca. Pelagibacter* sp. HTCC7211 (α-Proteobacteria)	APT [WP_008544956]	Y	Y
*Ca. Pelagibacter ubique* HTCC1062 (α-Proteobacteria)	APT [WP_011282223]	Y	Y
*Pseudomonas aeruginosa* PAO1 (γ-Proteobacteria)	APT01 [NP_250378]	Y	N
*Pseudomonas aeruginosa* PAO1 (γ-Proteobacteria)	APT02 [NP_253462]	T	Y
*Rhodothermus marinus* (Rhodothermota)	APT01 [WP_012844841]	Y	∗
*Rhodothermus marinus* (Rhodothermota)	APT02 [WP_012844658]	Y	Y
*Sulfobacillus acidophilus* (Bacillota, Clostridia)	APT01 [AEJ41924]	Y	N
*Sulfobacillus acidophilus* (Bacillota, Clostridia)	APT02 [AEJ39917]	Y	Y
*Thermoanaerobacter brockii* (Bacillota, Clostridia)	APT [WP_003867984]	Y	N
*Thermobrachium celere* (Bacillota, Clostridia)	APT [CDF58371]	Y	N
*Thermodesulfobacterium thermophilum* (Thermodesulfobacteriota)	APT01 [WP_022855228]	Y	N
*Thermodesulfobacterium thermophilum* (Thermodesulfobacteriota)	APT02 [WP_022855378]	Y	Y
*Thermodesulfovibrio yellowstonii* (Nitrospirota)	APT01 [ACI21680]	Y	N
*Thermodesulfovibrio yellowstonii* (Nitrospirota)	APT02 [ACI21563]	N	Y
*Thermosyntropha lipolytica* (Bacillota, Clostridia)	APT01 [SHG87240]	Y	N
*Thermosyntropha lipolytica* (Bacillota, Clostridia)	APT02 [SHG61007]	Y	Y
*Thermotoga maritima* (Thermotogota)	APT [AGL49579]	Y	N
*Thermus thermophilus* (Deinococcota)	APT [WP_011172918]	Y	Y
*Arabidopsis thaliana* (Streptophyta)	SpdSyn [Q9ZUB3]	Y	N
*Arabidopsis thaliana* (Streptophyta)	SpmSyn [BAH19534]	Y	Y
*Arabidopsis thaliana* (Streptophyta)	TspmSyn [OAO96167]	T	T
*Chlamydomonas reinhardtii* (Chlorophyta)	SpdSyn [XP_001702843]	Y	N
*Chlamydomonas reinhardtii* (Chlorophyta)	TspmSyn [ADF43120]	Y	Y
*Homo sapiens* (Metazoa)	SpdSyn [NP_003123]	Y	N
*Homo sapiens* (Metazoa)	SpmSyn [CAA88921]	T	T

Aminopropyltransferase activities determined in *Escherichia coli* BL21*speG* and BL21*speE*. The asterisk indicates APTs that produce Spm/Tspm in *E. coli* BL21*speE* but not in BL21*speG*.

Abbreviations: APT, aminopropyltransferase; N, no activity; SpdSyn, spermidine synthase; SpmSyn, spermine synthase, TspmSyn, thermospermine synthase; T, trace activity; Y, activity.

To determine whether the APT homologs that did not produce Spm/Tspm were functional SpdSyns, all genes were then expressed in a SpdSyn gene deletion strain (Δ*speE*) of *E. coli* BL21, which is devoid of Spd. The EICs for tribenzoylated Spd (m/z 458:459) and tetrabenzoylated Spm/Tspm from the cell extracts of BL21*speE* expressing the different APT genes are shown in Figs. S1–S3. All APTs that did not produce Spm/Tspm in BL21*speG* produced Spd in BL21*speE*, indicating SpdSyn activity (Table 1). Three of these SpdSyns were singleton APTs encoded by thermophilic species found previously to accumulate Spm/Tspm (30): *Thermotoga* *maritima*, *Thermoanaerobacter brockii*, and *Thermobrachium* *celere*. However, in the study of Hosoya *et al.* (30), each species was grown at temperatures of at least 60 °C, which may be required for Spm/Tspm synthase activity in those species. Notably, all Spm/Tspm synthases except those from *Pseudomonas aeruginosa* PAO1 and *Thermodesulfovibrio* *yellowstonii* produced Spm/Tspm in BL21*speE*, that is, they were able to synthesize Spm/Tspm from Put. This indicates that the Spm/Tspm synthases synthesize Spd from Put and then synthesize Spm/Tspm from Spd. This was also the case for the eukaryotic *A. thaliana* SpmSyn and *C. reinhardtii* TspmSyn but to a lesser degree for the *H. sapiens* SpmSyn and *A. thaliana* TspmSyn (Fig. S1). Therefore, the Spm/Tspm synthases do not necessarily require a dedicated partner SpdSyn to be able to produce Spm/Tspm from Put, except for those of *Pseudomonas aeruginosa* PAO1 and *Thermodesulfovibrio* *yellowstonii*.

**Figure 4:**
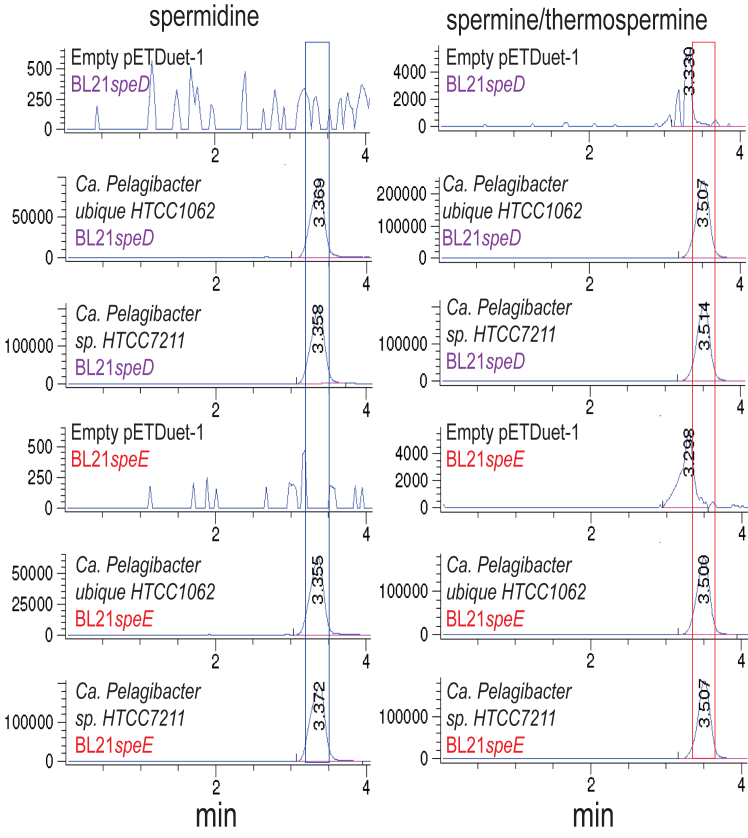
**Expression of *Ca.pelagibacter* species *S*-adenosylmethionine decarboxylase-aminopropyltransferase fusion proteins genes in *Escherichia coli* BL21*speD* and BL21*speE*.** Shown are the extracted ion chromatograms for tribenzoylated Spd (EIC = 457.94:498.94) and tetrabenzoylated Spm/Tspm (EIC = 619.02:620.02). The *blue* box outlines the position of Spd, and the *red* box outlines the position of Spm/Tspm detected by LC-MS. All genes were expressed from pETDuet-1. No Spd or Spm/Tspm is synthesized in either BL21*speD* or BL21*speE* with an empty pETDuet-1 expression vector. All strains were grown and processed in parallel.

Of the singleton APTs, the proteins from *Oceanithermus* *profundus* and *Thermus thermophilus* produced Spm/Tspm in BL21*speE*, that is, they are able to produce Spm/Tspm from Put. The singleton APTs from *Thermobrachium* *celere*, *Thermoanaerobacter brockii*, and *Thermotoga* *maritima* produce Spd but not Spm/Tspm in BL21*speE*. In contrast, the APT from *Leptospirillum* *ferrodiazotrophum* and APT01 from *Rhodothermus* *marinus*, which did not produce Spm/Tspm in BL21*speG*, generated detectable amounts of Spm/Tspm in BL21*speE*, which suggests that high levels of Spd in BL21*speG* may inhibit Spm/Tspm synthase activity of these APTs. The AdoMetDC-APT fusion proteins encoded by *Ca.* *Pelagibacter* *ubique* HTCC1062 and *Ca. Pelagibacter* sp. HTCC7211 produce Spm/Tspm when expressed in BL21*speE* (Fig. 4). They produce Spd when expressed in an AdoMetDC gene deletion strain (Δ*speD*) of BL21, confirming that the N-terminal AdoMetDC domain is functional (Fig. 4).

To determine whether each bacterial tetraamine (Spm/Tspm) synthase was specifically a Spm or Tspm synthase, we then developed an LC-MS/MS approach that chromatographically separated tetrabenzoylated Spm and Tspm. Using these conditions, commercially obtained Tspm and Spm eluted at 10.92 and 11.63 min, respectively (Fig. S4), and multiple reaction monitoring (MRM) mode was used to detect the tetrabenzoylated Spm and Tspm having the same MS transitions as they eluted off from the HPLC column at different retention times. We monitored the following transitions in MRM positive polarity mode, 619.228/497.2, 619.228/162, and 619.228/77 for both tetrabenzoylated Spm and Tspm. The quantifier ion was 619.228/497.2 while 619.228/162 and 619.228/77 were used as the qualifier ions. The quantifier ion was used to determine the analyte response using the peak area calculation, while the qualifier ions were used to help identify the analyte.

Analysis by LC-MS/MS of the eukaryotic Spm/Tspm synthases expressed in BL21*speG* indicated that the eukaryotic *H. sapiens* and *A. thaliana* SpmSyns are highly specific and do not produce any detectable Tspm (Table 2). The *C. reinhardtii* TspmSyn (Acl5) is also highly specific, whereas the *A. thaliana* TspmSyn (Acl5) produces a small amount of Spm in addition to Tspm. Detectable Spm was identified in the BL21*speG* strain containing an empty expression vector. It represented approximately 0.035% (area under the peak) of the Spm produced by expression of the *H. sapiens* SpmSyn. To assess whether the low level of Spm in the control BL21*speG* strain represented contamination or endogenous production of Spm by *E. coli*, we analyzed Tspm and Spm levels in BL21, BL21*speE*, and BL21*speG* expressing the empty pETDuet-1 plasmid (Table 3). A small amount of Tspm and approximately twice as much Spm was detected in BL21*speG*, whereas no Tspm or Spm was detected in BL21*speE*, indicating that the native *E. coli* SpdSyn produces a previously unnoticed low level of Spm and an even lower level of Tspm. The background level of Spm in the parental *E. coli* BL21 strain represents approximately 0.01% of the amount of Spm produced by the *H. sapiens* SpmSyn in BL21*speG* (Table 3).

**Table 2:** LC-MS/MS analysis of thermospermine and spermine production by validated aminopropyltransferases in *Escherichia coli* BL21*speG*

Aminopropyltransferase [GenBank protein acc. no]	Synthase activity	AUP 10.92 min (Tspm)	AUP 11.63 min (Spm)
Empty pETDuet-1	None	ND	5.21 × 10^4^
*Homo sapiens* SpdSyn [NP_003123]	Spermidine	ND	3.55 × 10^4^
*H. sapiens* SpmSyn [CAA88921]	Spermine	ND	1.48 × 10^8^
*Arabidopsis thaliana* SpdSyn1 [Q9ZUB3]	Spermidine	ND	9.49 × 10^3^
*A. thaliana* SpmSyn [BAH19534]	Spermine	ND	1.26 × 10^7^
*A. thaliana* Acl5 [OAO96167]	Thermospermine	3.79 × 10^7^	4.32 × 10^5^
*Chlamydomonas reinhardtii* SpdSyn [XP_001702843]	Spermidine	ND	5.79 × 10^4^
*C. reinhardtii* Acl5 [ADF43120]	Thermospermine	3.54 × 10^8^	ND

All genes expressed from pETDuet-1.

Abbreviations: Acl5, Acaulis 5/thermospermine synthase; AUP, area under the peak with elution time; ND, not detected; SpdSyn, spermidine synthase; Spm, spermine; SpmSyn, spermine synthase; Tspm, thermospermine.

**Table 3:** LC-MS/MS analysis of background thermospermine and spermine levels detected in different *E. coli* BL21 strains

Aminopropyltransferase and *Escherichia coli* BL21 strain	Synthase activity	AUP 10.92 min (Tspm)	AUP 11.63 min (Spm)
Empty pETDuet-1 in BL21	Native spermidine	ND	1.93 × 10^4^
Empty pETDuet-1 in BL21*speE*	None	ND	ND
Empty pETDuet-1 in BL21*speG*	Native spermidine	1.21 × 10^4^	2.56 × 10^4^
*Homo sapiens* SpmSyn [CAA88921]	Spermine	ND	1.88 × 10^8^
*Chlamydomonas reinhardtii* Acl5 [ADF43120]	Thermospermine	2.85 × 10^8^	ND

Tspm (thermospermine), Spm (spermine), SpdSyn (spermidine synthase), SpmSyn (spermine synthase), Acl5 (Acaulis 5/thermospermine synthase), AUP (area under the peak with elution time) from parent ion/daughter ion 619.228/497.200 (619.228 = tetrabenzoylated spermine/thermospermine). Heterologous genes expressed from pETDuet-1.

Abbreviations: ND, not detected; *speE*, spermidine synthase; *speG*, spermidine *N*^1^-acetyltransferase.

Bacterial Spm/Tspm synthases were expressed in either BL21*speE* or BL21*speG* according to which strain allowed the greater accumulation of Spm/Tspm. Using an arbitrary criterion of at least 10-fold more production of either Spm or Tspm, most of the bacterial Spm/Tspm synthases were either specifically Spm or Tspm synthases. The specific Tspm synthases were identified from *Arthrospira* *platensis*, *Thermodesulfobacterium* *thermophilum*, *Thermodesulfovibrio* *yellowstonii*, *Dictyoglomus* *thermophilum*, *Fimbriimonas ginsengisoli*, *Thermus thermophilus*, and surprisingly, the opportunistic human pathogen *Pseudomonas aeruginosa* PAO1 (Table 4). Specific SpmSyns were identified from *Geobacillus* *stearothermophilus*, *Sulfobacillus* *acidophilus*, *Desulfoarculus baarsii*, *Rhodothermus* *marinus* APT01 (which is primarily a SpdSyn), *Desulfosporosinus* *orientis*, *Heliorestis* *convoluta*, *Thermosyntropha lipolytica*, and *Oceanithermus* *profundus* (Table 4). APTs from *Rhodothermus* *marinus* (APT02), *Ca*. *Pelagibacter* sp. HTCC7211, and *Ca*. *P. ubique* HTCC1062 could be considered as bifunctional Spm/Tspm synthases.

**Table 4:** LC-MS/MS detection of thermospermine and spermine production by bacterial aminopropyltransferases in *Escherichia coli* BL21 strains

Species	Aminopropyltransferase	AUP Tspm	AUP Spm	Synthase activity
		10.92 min	11.63 min	
Group 1				
Empty pETDuet-1 in BL21	Endogenous SpdSyn	ND	1.93 × 10^4^	Spd
Empty pETDuet-1 in BL21*speE*	None	ND	ND	None
Empty pETDuet-1 in BL21*speG*	Endogenous SpdSyn	ND	2.56 × 10^4^	Spd
*Homo sapiens* in BL21*speG*	SpmSyn [CAA88921]	ND	1.88 × 10^8^	Spm
*Chlamydomonas reinhardtii* in BL21*speG*	Acl5 [ADF43120]	2.85 × 10^8^	ND	Tspm
Group 2 (in BL21*speG*)				
Empty pETDuet-1	Endogenous SpdSyn	ND	2.73 × 10^5^	Spd
*Geobacillus stearothermophilus*	APT01 [WP_049624640]	ND	1.47 × 10^5^	Spd
*Geobacillus stearothermophilus*	APT02 [WP_033014118]	2.90 × 10^4^	1.12 × 10^9^	Spm
*Sulfobacillus acidophilus*	APT01 [AEJ41924]	ND	1.03 × 10^5^	Spd
*Sulfobacillus acidophilus*	APT02 [AEJ39917]	ND	1.75 × 10^8^	Spm
*Arthrospira platensis*	APT01 [BAI92260]	2.84 × 10^6^	1.03 × 10^5^	Tspm
*Arthrospira platensis*	APT02 [BAI90257]	1.26 × 10^5^	2.30 × 10^5^	Spd
Group 3 (in BL21*speG*)				
Empty pETDuet-1	Endogenous SpdSyn	ND	2.91 × 10^4^	Spd
*Thermodesulfobacterium thermophilum*	APT02 [WP_022855378]	1.37 × 10^8^	4.11 × 10^5^	Tspm
*Thermodesulfovibrio yellowstonii*	APT02 [ACI21563]	9.50 × 10^7^	3.28 × 10^5^	Tspm
*Pseudomonas aeruginosa* PAO1	APT02 [NP_253462]	3.72 × 10^6^	4.82 × 10^4^	Tspm
Group 4 (in BL21*speG*)				
*Desulfarculus baarsii*	APT01 [WP_013259899]	ND	1.45 × 10^9^	Spm
*Dictyoglomus thermophilum*	APT02 [WP_012547113]	1.26 × 10^7^	1.04 × 10^5^	Tspm
*Fimbriimonas ginsengisoli*	APT01 [WP_025227485]	9.26 × 10^8^	3.19 × 10^6^	Tspm
*Rhodothermus marinus*	APT01 [WP_012844841]	3.96 × 10^4^	3.33 × 10^8^	Spm
*Rhodothermus marinus*	APT02 [WP_012844658]	2.71 × 10^8^	1.46 × 10^9^	Spm>Tspm
Group 5 (in BL21*speE*)				
Empty pETDuet-1	None	ND	3.19 × 10^4^	Spd
*Desulfosporosinus orientis*	APT02 [WP_014182917]	1.06 × 10^4^	5.55 × 10^8^	Spm
*Heliorestis convoluta*	APT02 [WP_153723875]	ND	8.54 × 10^8^	Spm
*Thermosyntropha lipolytica*	APT01 [SHG87240]	4.90 × 10^4^	3.03 × 10^5^	Spm>Tspm
*Thermosyntropha lipolytica*	APT02 [SHG61007]	ND	5.28 × 10^8^	Spm
*Leptospirillum ferrodiazotrophum*	Single APT [EES53973]	1.68 × 10^5^	9.05 × 10^6^	Spm
Group 6 (in BL21*speG*)				
Empty pETDuet-1	Endogenous SpdSyn	ND	7.88 × 10^3^	Spd
*Thermus thermophilus*	Single APT [WP_011172918]	8.23 × 10^6^	1.63 × 10^5^	Tspm>>Spm
*Oceanithermus profundus*	Single APT [WP_013456826]	3.87 × 10^3^	6.80 × 10^7^	Spm
*Ca. Pelagibacter* sp. HTCC7211	Single APT [WP_008544956]	4.95 × 10^7^	7.92 × 10^7^	Spm/Tspm
*Ca. Pelagibacter ubique* HTCC1062	Single APT [WP_011282223]	4.18 × 10^7^	8.07 × 10^7^	Spm/Tspm

The area under the peak (AUP) for the tetrabenzoylated tetraamine 619.228/497.200 daughter ion is provided for thermospermine (elutes at 10.92 min) and spermine (11.52 min). Each group of *E. coli* strains were grown and analyzed by LC-MS/MS independently; within groups, all strains were grown and analyzed in parallel.

Abbreviations: APT, aminopropyltransferase; Spd, spermidine; *speE*, spermidine synthase; *speG*, spermidine *N*-acetyltransferase; Spm, spermine; Tspm, thermospermine.

### Production of norspermine and N^1^-aminopropylhomospermidine

SpmSyns transfer an aminopropyl group from dcAdoMet (Fig. 1*A*) to the *N*^8^-(aminobutyl) side of Spd to form Spm, whereas TspmSyns transfer the aminopropyl group to the *N*^1^-(aminopropyl) side of Spd to form Tspm (Fig. 1*B*). The symmetrical triamines norspermidine (Nspd) and homospermidine (Hspd) consist of only aminopropyl or aminobutyl moieties, respectively (Fig. 1*C*). We sought to determine whether the SpmSyns and TspmSyns would prefer Hspd or Nspd as substrates when expressed in the *E. coli* Spd-devoid BL21*speE*. Transformed strains were grown in polyamine-free M9 medium with added 0.5 mM Nspd or Hspd. Relative uptake efficiencies of each polyamine were not determined. The products of *N*-aminopropylation of Nspd and Hspd are norspermine (Nspm) and *N*^1^-aminopropylhomospermidine (*N*^1^-APHspd), respectively (Fig. 1*C*). Benzoylated polyamines from cell extracts were analyzed by LC-MS, and the EICs for tetrabenzoylated Nspm (m/z = 605) and tetrabenzoylated *N*^1^-APHspd (m/z = 634) are shown in Figs. S5–S7. The eukaryotic SpmSyns from *A. thaliana* and *H. sapiens* produced *N*^1^-APHspd but not Nspm, emphasizing their strict specificity for aminopropylating an aminobutyl but not an aminopropyl moiety (Table 5). Bacterial SpmSyns are also specific for aminobutyl moieties except for the APT from *Oceanithermus* *profundus*, which produced Nspm at a level of approximately 10% that of *N*^1^-APHspd. In contrast, the substrate specificity of TspmSyns is more complex, with the TspmSyns of *Dictyoglomus thermophilum* and *Pseudomonas aeruginosa* PAO1 being strictly selective for Nspd over Hspd as substrates, whereas the other TspmSyns exhibit activity with both triamines. The TspmSyns from *Arthrospira plantensis, Thermus thermophilus*, and *Fimbriimonas* *ginsengisoli* are equally or more active with Hspd than Nspd. The singleton APT from *Leptospirillum* *ferrodiazotrophum* produced *N*^1^-APHspd, consistent with its SpmSyn activity. These data suggest that all investigated TspmSyns are potentially NspmSyns, if Nspd is present as a substrate.

**Table 5:** Norspermidine and *N*^1^-aminopropylhomospermidine synthase activities of aminopropyltransferases

Species (Phylum)	Protein [Genbank acc.no.]	Nspm	*N*^1^APHspd
*Arabidopsis thaliana* (Streptophyta)	TspmSyn [OAO96167]	Y	T
*Arabidopsis thaliana* (Streptophyta)	SpmSyn [BAH19534]	N	Y
*Arthrospira platensis* (Cyanobacteriota)	TspmSyn [BAI92260]	Y	Y
*Chlamydomonas reinhardtii* (Chlorophyta)	TspmSyn [ADF43120]	Y	T
*Desulfarculus baarsii* (Thermodesulfobacteriota)	SpmSyn [WP_013259899]	N	Y
*Desulfosporosinus orientis* (Bacillota, Clostridia)	SpmSyn [WP_014182917]	N	Y
*Dictyoglomus thermophilum* (Dictyoglomota)	TspmSyn[WP_012547113]	Y	N
*Fimbriimonas ginsengisoli* (Armatimonadota)	TspmSyn [WP_025227485]	Y	Y
*Geobacillus stearothermophilus* (Bacillota, Bacilli)	SpmSyn [WP_033014118]	N	Y
*Homo sapiens* (Metazoa)	SpmSyn [Q9ZUB3]	N	Y
*Heliorestis convoluta* (Bacillota, Clostridia)	SpmSyn [WP_153723875]	N	Y
*Leptospirillum ferrodiazotrophum* (Nitrospirota)	SpdSyn [EES53973]	N	Y
*Oceanithermus profundus* (Deinococcota)	SpmSyn [WP_013456826]	Y	Y
*Pseudomonas aeruginosa* PAO1 (γ-Proteobacteria)	TspmSyn [NP_253462]	Y	N
*Rhodothermus marinus* (Rhodothermota)	SpmSyn [WP_012844841]	N	Y
*Rhodothermus marinus* (Rhodothermota)	TspmSyn [WP_012844658]	N	Y
*Sulfobacillus acidophilus* (Bacillota, Clostridia)	SpmSyn [AEJ39917]	N	Y
*Thermodesulfob*. *thermophilum* (Thermodesulfobacteriota)	TspmSyn [WP_022855378]	Y	Y
*Thermodesulfovibrio yellowstonii* (Nitrospirota)	TspmSyn [ACI21563]	Y	Y
*Thermosyntropha lipolytica* (Bacillota, Clostridia)	SpmSyn [SHG61007]	N	Y
*Thermus thermophilus* (Deinococcota)	APagmSyn [WP_011172918]	Y	Y
*Arabidopsis thaliana* (Streptophyta)	SpmSyn [BAH19534]	N	Y
*Arabidopsis thaliana* (Streptophyta)	TspmSyn [OAO96167]	Y	T
*Chlamydomonas reinhardtii* (Chlorophyta)	TspmSyn [ADF43120]	Y	T
*Homo sapiens* (Metazoa)	SpmSyn [CAA88921]	N	Y

Aminopropyltransferase genes were expressed from pETDuet-1 in *E. coli* BL21*speE* grown with 0.5 mM norspermidine (Nspd) or homospermidine (Hspd). Based on data derived from Figs. S5–S7.

Abbreviations: APagmSyn, *N*^1^-aminopropylagmatine synthase; N, no activity; SpmSyn, spermidine synthase; T, trace activity; TspmSyn, thermospermine synthase; Y, synthase activity.

### Production of norspermidine from 1,3-diaminopropane

**Figure 5:**
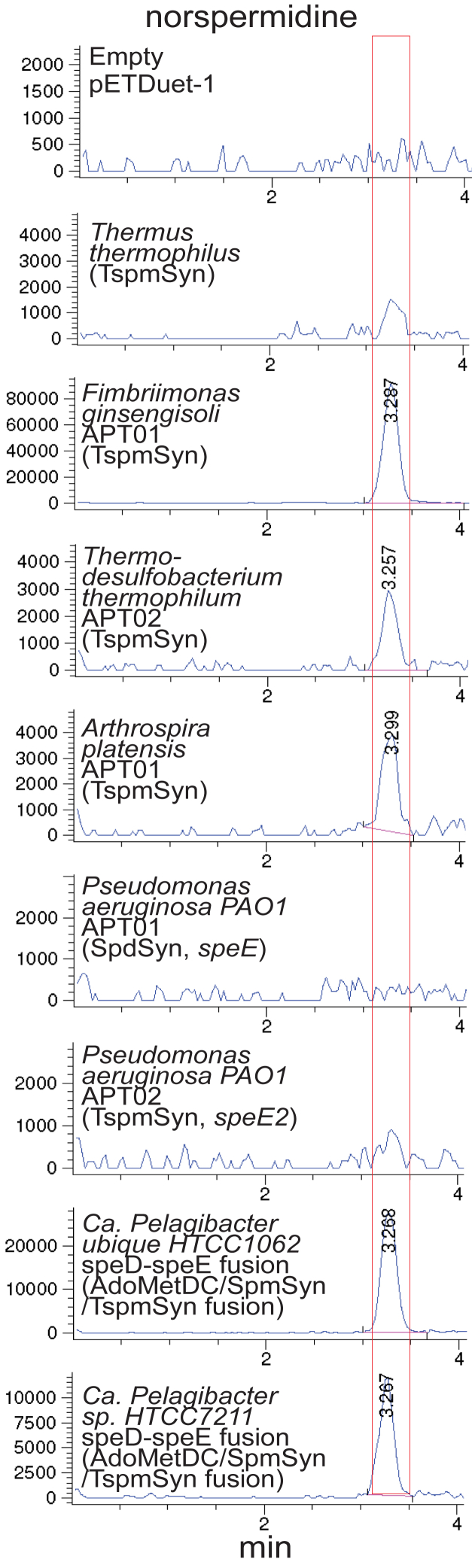
**Norspermidine production by aminopropyltransferases expressed in *Escherichia coli* BL21*speE* grown with 1.0 mM 1,3-diaminopropane.** Shown are the LC-MS extracted ion chromatograms for tribenzoylated Nspd (EIC = 443.92:444.92). The *red* box outlines the position of Nspd detected by LC-MS. All genes were expressed from pETDuet-1. No Nspd, Spd, or Spm/Tspm is synthesized in either BL21*speD* or BL21*speE* with an empty pETDuet-1 expression vector. All strains were grown and processed in parallel.

*Pseudomonas aeruginosa* PAO1 strain has been reported to produce Nspd from 1,3-diaminopropane (Dap) (Fig. 1*C*) and that Nspd production may participate in resistance to antibiotics (40). The gene suggested as the NspdSyn is PA4774 (*speE2*) (40), which is the gene we identified as encoding a TspmSyn. It is immediately downstream of a gene encoding a class 1b AdoMetDC (PA4773, SpeD2, AE004891, 160 aa). In addition to this class 1b AdoMetDC (38), PAO1 also encodes a class 1a AdoMetDC (PA0654, SpeD, WP_003101641, 264 aa). The *P*. *aeruginosa* PAO1 SpdSyn and TspmSyn exhibit 60% and 36% a.a. identity to the *E. coli* K12 SpdSyn, respectively. Because of the importance of *P. aeruginosa* as an opportunistic human pathogen, we examined whether either of the two APTs of *P. aeruginosa* were able to produce Nspd from Dap. TspmSyns aminopropylate aminopropyl moieties, so we also analyzed a selection of other bacterial TspmSyns. The genes were expressed in Spd-devoid BL21*speE* grown in M9 medium containing 1.0 mM Dap. TspmSyn of *Fimbriimonas* *ginsengisoli*, *Ca. P. ubique* HTCC1062, and *Ca. Pelagibacter* sp. HTCC7211 produced relatively large quantities of Nspd from Dap (Fig. 5). These TspmSyns are therefore potentially NspdSyns if the substrate Dap is present. Smaller amounts were produced by the TspmSyns of *Thermodesulfobacterium* *thermophilum* and *Arthrospira* *platensis* but no detectable Nspd was produced by the APT of *Thermus thermophilus* or the SpdSyn or TspmSyn of *P. aeruginosa* PAO1. It is possible that in *P. aeruginosa* PAO1, the Tspm produced by PA 4774 (TspmSyn, SpeE2) is oxidized by an unknown oxidase to Nspd, as observed with the vascular plant *Selaginella lepidophylla* (41) or by converted to Nspd by a dehydrogenase.

### Most ***spermidine*** synthases are N^1^-aminopropylagmatine synthases

**Figure 6:**
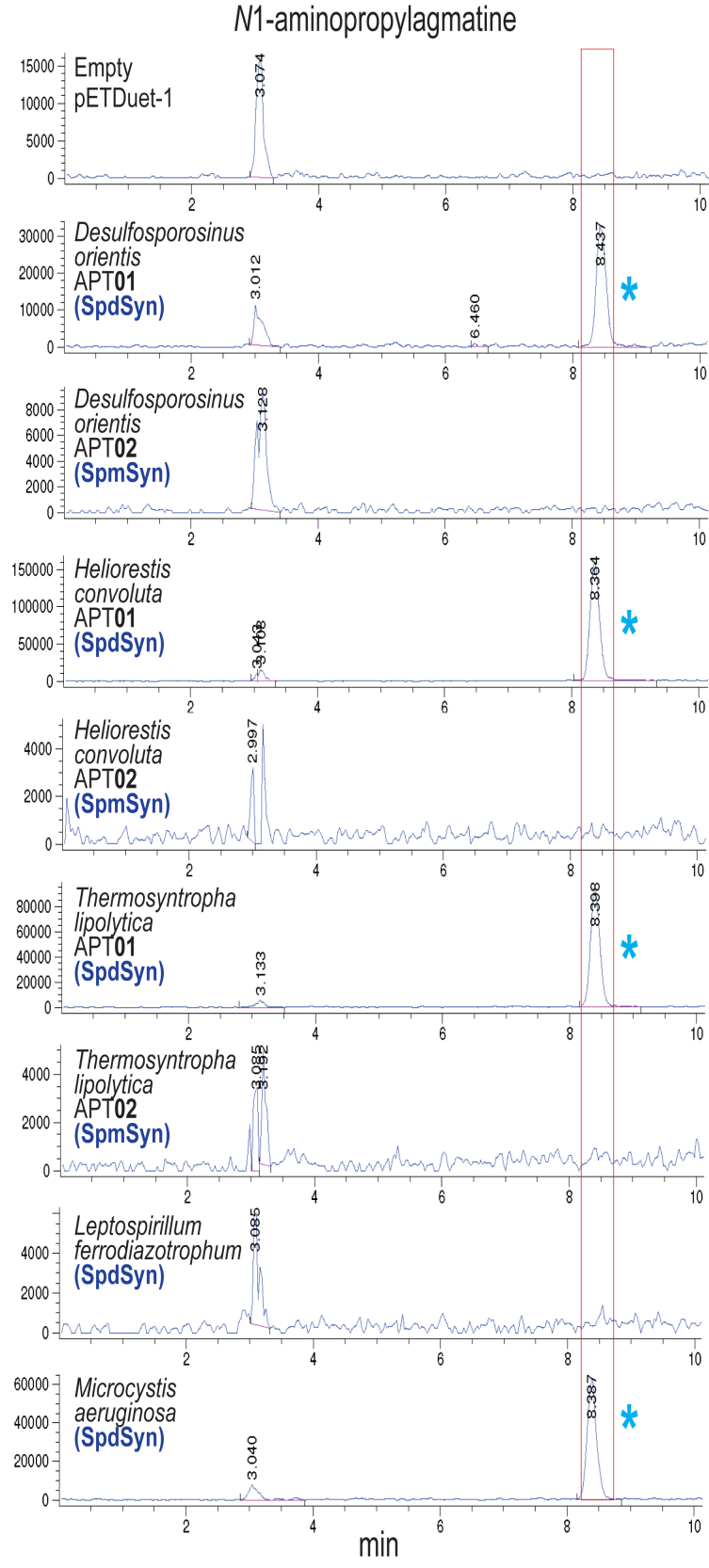
***N***^**1**^**-aminopropylagmatine production by aminopropyltransferases expressed in *Escherichia coli* BL21*speG*.** Shown are the LC-MS extracted ion chromatograms for tetrabenzoylated *N*^1^-aminopropylagmatine (*N*^1^-APAgm, EIC = 603.98:604.98). The *red box* outlines the position of *N*^1^-APAgm, and the *blue* asterisk indicates the presence of a peak for *N*^1^-APAgm. Genes were expressed from pETDuet-1. All strains were grown and processed in parallel.

We have considered the aminopropylation of Put to form Spd and of Spd to form either Spm or Tspm. Another form of aminopropylation has been described for the bacterium *Thermus thermophilus* (33) and the archaeon *Thermococcus kodakarensis* (42): the aminopropylation of agmatine (Agm) to form *N*^1^-aminopropylagmatine (*N*^1^-APAgm) (Fig. 1*D*). In this pathway, *N*^1^-APAgm is converted to Spd by an agmatinase homolog (*N*^1^-aminopropylagmatinase). The single APT of *Thermus thermophilus* aminopropylates both Agm and Spd (33). We noticed that some of the SpdSyns we expressed in BL21*speG* produced a peak corresponding to tetrabenzoylated *N*^1^-APAgm (m/z 604.2) (Fig. 6). For example, *Desulfosporosinus* *orientis*, *Heliorestis* *convoluta*, and *Thermosynthropha lipolytica*, which encode two APTs each, the SpdSyn but not the SpmSyn produced *N*^1^-APAgm in BL21*speG*. The SpdSyn of *Microcystis aeruginosa* also produced *N*^1^-APAgm.

**Figure 7:**
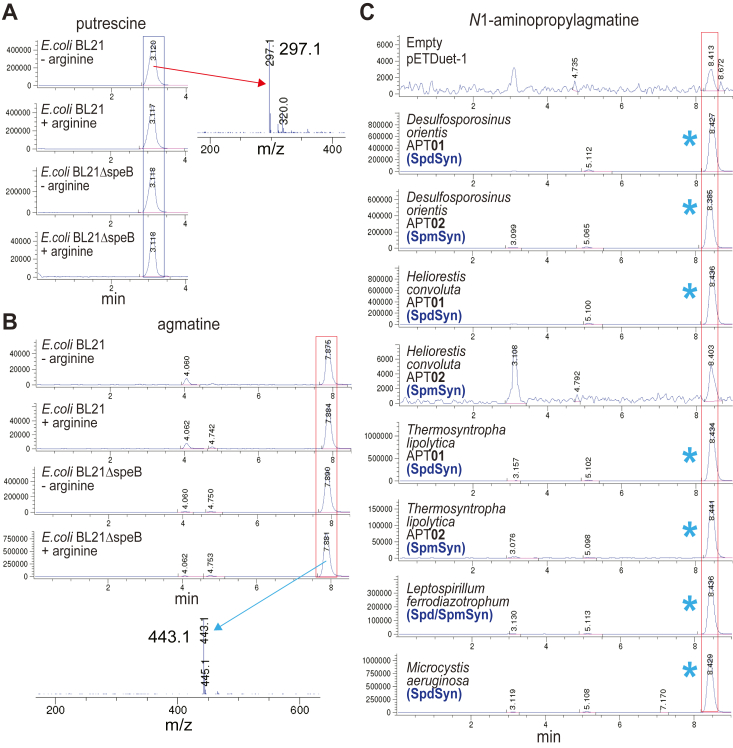
**Expression of aminopropyltransferases in *Escherichia coli* BL21*speB*.** All genes were expressed from pETDuet-1. *A*, LC-MS extracted ion chromatograms for dibenzoylated Put (EIC = 296.85:297.85). The *blue* box outlines the position of Put. Cultures of *E. coli* BL21 and BL21*speB* were grown with ± 300 μM L-arginine and expressed the empty pETDuet-1 plasmid. All strains were grown and processed in parallel. The inset shows the mass spectrum for the dibenzoylated putrescine peak. *B*, LC-MS extracted ion chromatograms for tribenzoylated Agm (EIC = 442.9:443.9). The *red* box outlines the position of Agm. Cultures of *E. coli* BL21 and BL21*speB* were grown with ± 300 μM L-arginine and expressed the empty pETDuet-1 plasmid. All strains were grown and processed in parallel. The inset shows the mass spectrum for the tribenzoylated agmatine peak. *C*, LC-MS extracted ion chromatograms for tetrabenzoylated *N*^1^-aminopropylagmatine (*N*^1^-APAgm, EIC = 603.98:604.98) in the cell extracts of *E. coli* BL21*speB* expressing aminopropyltransferase genes from pETDuet-1 and grown with 300 μM L-arginine. The *red box* outlines the position *N*^1^-APAgm. All strains were grown and processed in parallel. The *blue* asterisk indicates the presence of peaks for *N*^1^-APAgm (m/z 604.4). Put, putrescine.

We decided to more fully explore the aminopropylation of Agm by the APTs described so far in our study. As a positive control, we included the *N*^1^-APAgm synthase from *Thermococcus kodakarensis*, and as negative controls, the SpdSyns of *E. coli*, *Bacillus subtilis* (43), and an uncultivated bacteriophage (3). To facilitate this analysis, by increasing Agm levels in *E. coli*, a gene deletion of agmatinase in BL21 was created (BL21Δ*speB*). Genes were expressed in *E. coli* BL21*speB* grown with 300 μM arginine, which partially represses flux through the arginine biosynthesis pathway, thereby reducing Put but increasing Agm levels (Fig. 7). Using this approach, Put decreased by 10-fold (Fig. 7*A*) and Agm levels were increased approximately 20-fold (Fig. 7*B*). Unexpectedly, we found that all SpdSyns except those from *P. aeruginosa* PAO1 and the uncultivated Caudovirales bacteriophage produced *N*^1^-APAgm (Figs. 7*C*, S8 and S9). Whereas the SpmSyns of *Desulfosporosinus* *orientis* and *Thermosynthropha lipolytica* did not produce *N*^1^-APAgm in BL21*speG*, they did produce *N*^1^-APAgm in BL21*speB* grown with 300 μM arginine, although less than produced by the corresponding SpdSyns. Overexpression of the *E. coli* SpdSyn produced only a relative trace of *N*^1^-APAgm compared to, for example, SpdSyn of *Bacillus subtilis*, which produced approximately as much *N*^1^-APAgm in this system as the *Thermococcus* *kodakarensis N*^1^-APAgm synthase. The *N*^1^-APAgm synthase activity of the *Bacillus subtilis* SpdSyn (SpeE) enzyme was entirely unexpected.

We reasoned that species encoding an *N*^1^-APAgmSyn should also encode an L-arginine decarboxylase to produce Agm. If the purpose of *N*^1^-APAgm production is to bypass Put in Spd biosynthesis, as is thought to be the case for *Thermus thermophilus* (33) and *T. kodakarensis* (42), the presence of L-ornithine decarboxylase, that converts ornithine directly to Put, would undermine that purpose. The genomes of the species encoding the APTs analyzed in the current study were interrogated for all the known forms of L-arginine and L-ornithine decarboxylases (44, 45). Most species encoding *N*^1^-APAgmSyn activity were found to encode only L-arginine decarboxylases, albeit from four different protein folds (Table S2). Consistent with the lack or low level of *N*^1^-APAgmSyn activity from the APTs of *P. aeruginosa* PAO1 and *E. coli*, these two species each encode L-arginine and L-ornithine decarboxylases. Exceptions are *Thermodesulfovibrio* *yellowstonii* and *Thermotoga* *maritima*, which encode *N*^1^-APAgmSyn activity but possess only L-ornithine decarboxylase homologs. The genome of *Oceanithermus* *profundus* does not appear to encode a recognizable L-arginine or L-ornithine homolog, which suggests a novel form of these enzymes remains to be discovered or else this organism would have to take up exogenous Agm or Put.

In contrast to the SpdSyn homologs, the Spm and Tspm synthases did not produce *N*^1^-APAgm except for the SpmSyns of *Desulfosporosinus* *orientis*, *Thermosyntropha lipolytica*, *Rhodothermus* *marinus*, and *Geobacillus stearophilus*. The first two of these did not produce *N*^1^-APAgm when expressed in BL21*speG* (Fig. 6). No TspmSyn produced *N*^1^-APAgm except the APT of *Thermus thermophilus*, which was originally identified as a bifunctional *N*^1^-APAgm synthase/TspmSyn (33). The production of *N*^1^-APAgm was confirmed using LC-high resolution mass spectrometry (LC-HRMS), and selected species are shown in Fig. S10.

### Biosynthesis of N^12^-guanidinothermospermine

Production of *N*^1^-APAgm by SpdSyn homologs raised the possibility that some bacterial species encoding TspmSyn might aminopropylate the aminopropyl side of *N*^1^-APAgm synthesized by the SpdSyn/*N*^1^-APAgmSyn to produce (*N*^1^-aminopropyl)- *N*^1^-APAgm, that is, *N*^12^-guanidinothermospermine (Fig. 1*D*) that might potentially then be hydrolyzed by a ureohydrolase to Tspm. We selected SpdSyn/*N*^1^-APAgmSyn and TspmSyn pairs encoded by *Arthrospira* *platensis*, *Dictyoglomus thermophilum*, *Fimbriimonas* *ginsengisoli*, and *Thermodesulfobacterium thermophilum* for co-expression in *E. coli* BL21*speB*.

**Figure 8:**
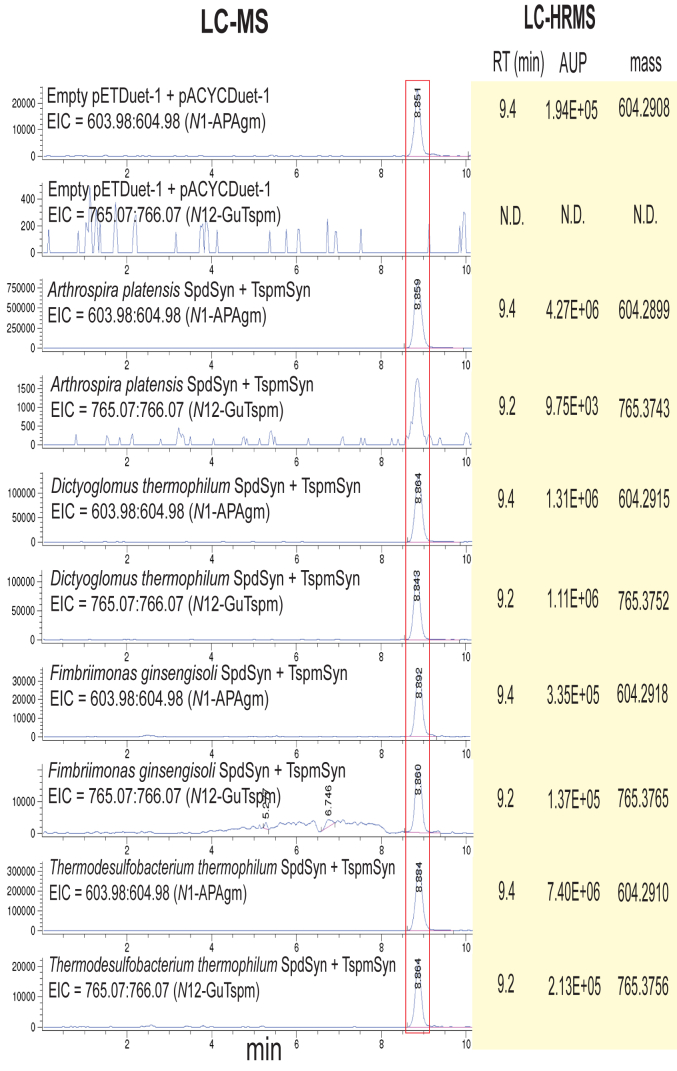
**LC-MS detection of *N***^**1**^**-aminopropylagmatine and *N***^**12**^**-guanidinothermospermine.** SpdSyn/*N*^1^-APAgmSyn in pETDuet-1 and TspmSyn in pACYCDuet-1 were coexpressed in *E. coli* BL21*speB* and grown in the presence of 3 mM L-arginine. The *left*-hand panel shows the extracted ion chromatograms from the LC-MS detection of tetrabenzoylated *N*^1^-aminopropylagmatine (*N*^1^-APAgm, EIC = 603.98:604.98) and pentabenzoylated *N*^12^-guanidinothermospermine (*N*^12^-GuTspm, EIC = 765.07:766.07). The peaks for *N*^1^-APAgm and *N*^12^-GuTspm elute at similar times and are highlighted by the *red box*. In an independent experiment, the same cultures were grown again for analysis by LC-high resolution MS. In the *right*-hand panel, the retention times, area under the peak (AUP), and accurate mass of tetrabenzoylated *N*^1^-APAgm and pentabenzoylated *N*^12^-GuTspm are shown for the corresponding cultures in the *left*-hand panel. N.D., not detected.

To further increase the level of Agm and decrease Put available for this reaction sequence, BL21*speB* was grown with 10-fold more L-arginine (3 mM) to repress flux from L-glutamate to L-ornithine. Under this growth condition, the native SpdSyn of *E. coli* BL21*speB* produced easily detectable levels of *N*^1^-APAgm, as the tetrabenzoylated form (Fig. 8*A*). However, with BL21*speB* expressing empty vectors, no *N*^12^-guanidinothermospermine was detected (pentabenzoylated form EIC = 765.07:766.07). Similarly, the APT pair from *Arthrospira* *platensis* did not produce detectable *N*^12^-guanidinothermospermine. In contrast, the other APT pairs produced easily detectable levels of *N*^12^-guanidinothermospermine, particularly so for *Dictyoglomus thermophilum*. The tetrabenzoylated *N*^1^-APAgm and pentabenzoylated *N*^12^-guanidinothermospermine eluted at the same time from the LC column, so the fraction eluting at 8.86 to 8.88 min contained the masses of both metabolites (Fig. S11). To further confirm the identities of tetrabenzoylated *N*^1^-APAgm and pentabenzoylated *N*^12^-guanidinothermospermine, the experiment was repeated, and benzoylated cell extracts were analyzed by LC-HRMS (Fig. 8*B*). The exact masses of tetrabenzoylated *N*^1^-APAgm and pentabenzoylated *N*^12^-guanidinothermospermine were detected.

### Prediction of aminopropyltransferase function from amino acid sequence

**Figure 9:**
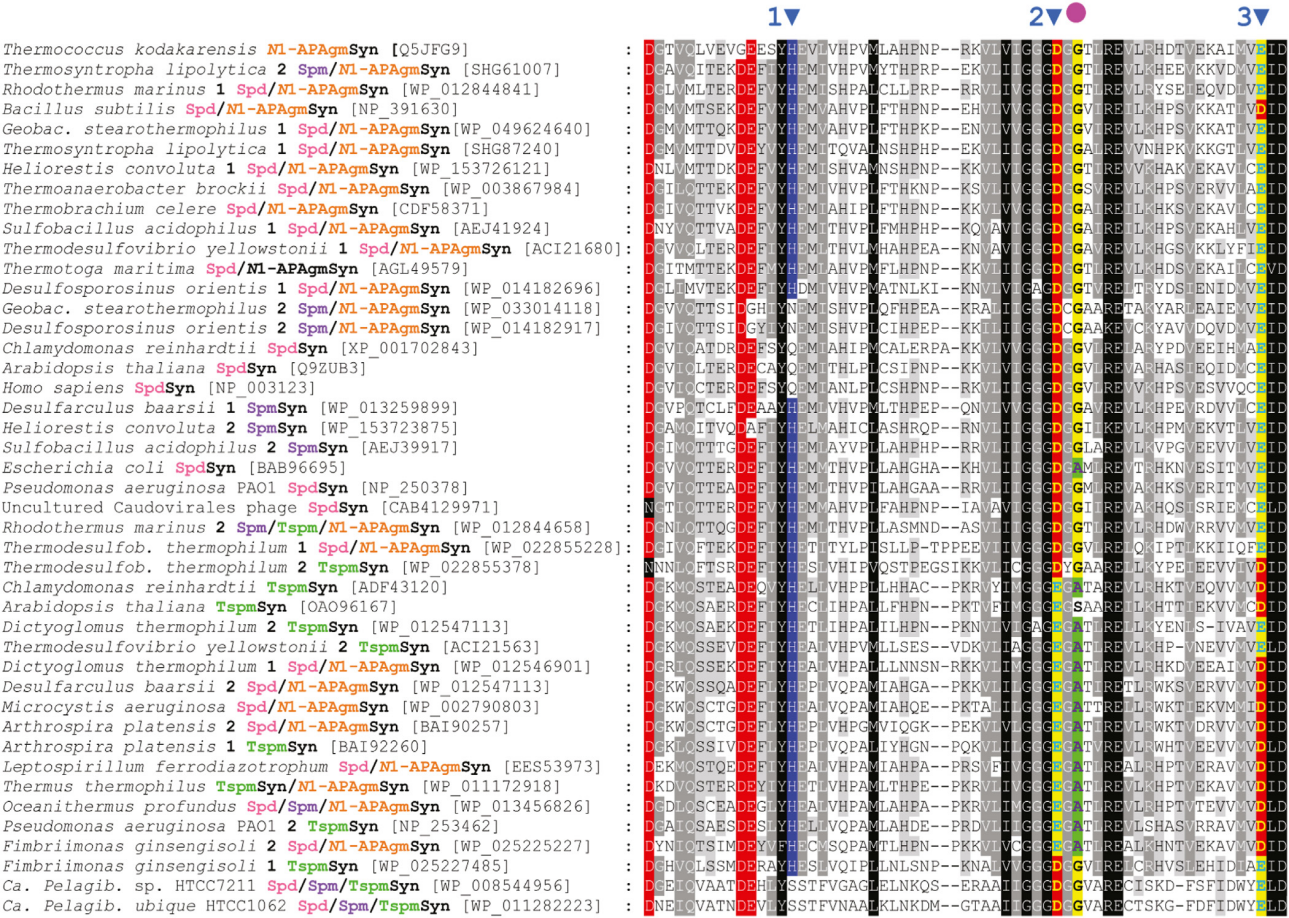
**Amino acid sequence alignment of the analyzed aminopropyltransferases.** The alignment covers the region corresponding to the *Escherichia coli* SpeE (SpdSyn) amino acid positions 50 to 110. *Blue arrows* indicate significant motifs. The *red circle* indicates the position 2 amino acids downstream of motif 2.

Currently, there is only one SpmSyn and one TspmSyn published X-ray crystal structure (6, 8), although structures exist for the substrate-flexible APTs of *Thermus thermophilus* (7) and the crenarchaeote *Pyrobaculum calidifontis* (46). Active site residues for the SpdSyn, SpmSyn, and TspmSyn proteins of the leguminous plant *Medicago trunculata* have been compared to identify key differences (8). APTs bind dcAdoMet and a polyamine substrate in the active site cleft. Three amino acids involved in binding dcAdoMet differentiate the plant TspmSyn from SpdSyn and SpmSyn (8). In the *M. trunculata* TspmSyn, these amino acids correspond to His85, Glu109, and Asp129 (marked as motifs 1, 2, & 3, respectively in Fig. 9). The Glu109 position (motif 2) is found in a highly conserved sequence hhhhGGG(D/E)G(G/A), where h represents a small hydrophobic residue. The presence of a Glu rather than an Asp in motif 2 was thought previously to discriminate TspmSyn from SpdSyn and SpmSyn (15, 16). Comparison of the APTs analyzed in our current study suggests that the His85 position in motif 1 is not informative. In contrast, all APTs from our study acting principally as SpmSyns possess an Asp in motif 2 and Glu in motif 3. Furthermore, the position 2 amino acids downstream of the Asp in motif 2 is always a Gly in SpmSyns (D(G/C)G). This pattern distinguishes SpmSyns from Spd/*N*^1^-APAgmSyns and TspmSyns, with the caveat that the SpmSyns in our study are phylogenetically skewed towards the Bacillota phylum. APTs exhibiting primarily TspmSyn activity possess a Glu in motif 2, except for the TspmSyns of *Fimbriimonas* *ginsengisoli* and *Thermodesulfobacterium* *thermophilum* that have an Asp. SpdSyn/*N*^1^-APAgmSyns are not distinguished by any of the motifs.

**Figure 10:**
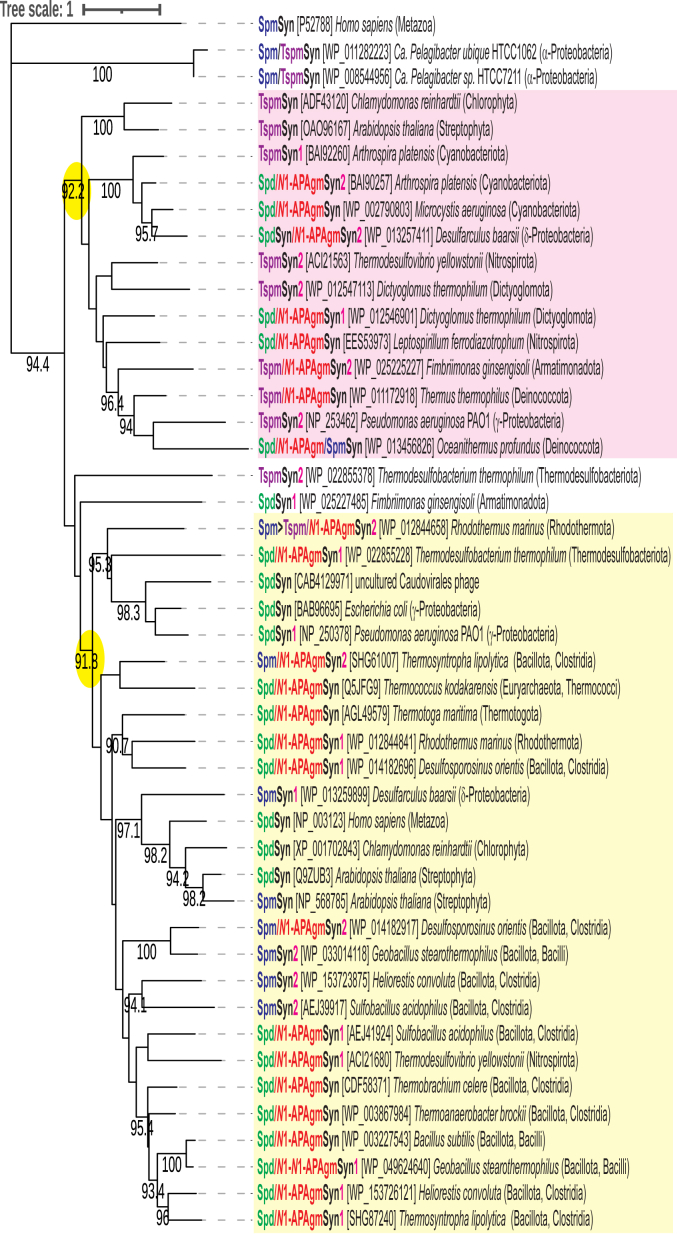
**Maximum likelihood phylogenetic tree of the analyzed aminopropyltransferases.** TspmSyn (thermospermine synthase), SpmSyn (spermine synthase), SpdSyn (spermidine synthase), Spd/*N*^1^-APAgmSyn (spermidine/*N*^1^-aminopropylagmatine synthase). Numbers after enzyme name indicate aminopropyltransferase pairs; no number indicates a singleton aminopropyltransferase. Phyla are indicated in parentheses after the species name. The *pink box* covers TspmSyns and Spd/*N*^1^-APAgmSyns; *yellow* box covers SpmSyns, SpdSyns, and Spd/*N*^1^-APAgmSyns. Numerical values represent percentage bootstrap support above 90% from 1000 ultrafast bootstrap analyses. The scale bar represents the average number of amino acid substitutions per site.

A phylogenetic tree of the APT proteins shows that there are two, well supported clades (Fig. 10). One clade consists of TspmSyns interspersed with Spd/*N*^1^-APAgmSyns, and the other consists of SpdSyns and Spd/*N*^1^-APAgmSyns interspersed with SpmSyns. For genomes encoding pairs of APTs, there are strong candidates for horizontal acquisition of one of the genes, for example, each member of the APT pairs of *Thermodesulfovibrio* *yellowstonii* and *Desulfarculus baarsii* are found in separate clades. In contrast, the two APTs of *Arthrospira* *platensis* and of *Dictyoglomus thermophilum* are found close together on the Maximum Likelihood tree, indicating gene duplication and neofunctionalization. The APT pairs of *Heliorestis* *convoluta*, *Geobacillus* *stearothermophilus*, and *Sulfobacillus* *acidophilus* are found in the same subclade. They may represent a common ancestral gene duplication and neofunctionalization followed by vertical divergence.

## Discussion

It has been proposed that bacteria do not encode SpmSyn (15); however, our study has functionally identified for the first time *bona fide* SpmSyns in bacteria. They are found in diverse phyla: *Geobacillus* *stearothermophilus*, *Sulfobacillus* *acidophilus*, *Desulfosporosinus* *orientis*, *Heliorestis* *convoluta*, and *Thermosynthropha lipolytica* (Bacillota phylum, formerly Firmicutes); *Rhodothermus* *marinus* (Rhodothermota); *Desulfoarculus baarsii* (Thermodesulfobacteriota); *Leptospirillum* *ferrodiazotrophum* (Nitrospirota), *Oceanithermus* *profundus* (Deinococcota), and *Ca. Pelagibacter* species (Pseudomonadota, class α-Proteobacteria). We have also functionally identified TspmSyns from equally diverse phyla: *Arthrospira* *platensis* (Cyanobacteriota); *Thermodesulfobacterium* *thermophilum* (Thermodesulfobacteriota); *Thermodesulfovibrio* *yellowstonii* (Nitrospirota); *Dictyoglomus thermophilum* (Dictyoglomota); *Fimbriimonas* *ginsengisoli* (Armatimonadota), *Ca. Pelagibacter* species (Pseudomonadota, class α-Proteobacteria), *Pseudomonas* *aeruginosa* PAO1 (class γ-Proteobacteria), and we have reconfirmed the *Thermus thermophilus* (Deinococcota) TspmSyn activity. It is notable that we did not identify a TspmSyn in the Bacillota phylum. However, there are some genomes encoding APTs with TspmSyn homology, for example, *Dethiobacter alkaliphilus* (Bacillota, Dethiobacteria) encodes a TspmSyn homolog (WP_264698086, 304 aa) and a Spd/*N*^1^-APAgmSyn homolog (WP_264697253, 276 aa). The APTs from *Ca. Pelagibacter* species standout because they are Spd/Spm/Tspm synthases fused at the N terminus to a functional AdoMetDC. Both SpmSyns and TspmSyns are found in thermophiles and also mesophiles.

All bacterial Spm and Tspm synthases were able to varying degrees to produce either Spm or Tspm from Put, with the exception of the TspmSyns from *P*. *aeruginosa* PAO1 and *Thermodesulfovibrio* *yellowstonii*. This would be consistent with the idea that Spm/Tspm synthases evolved from Spd/*N*^1^-APAgm synthases and retained SpdSyn function. A potential advantage of encoding distinct Spd/*N*^1^-APAgm synthases, and separate Spm/Tspm synthases that can synthesize Spm/Tspm from Put, in the same cell, is that Spm/Tspm biosynthesis could be uncoupled from Spd and even Put biosynthesis.

A universal feature of TspmSyns that we observed is their ability to synthesize Nspm from Nspd, implying that Nspm biosynthesis is an inherent characteristic of TspmSyns. That is to say, TspmSyns are *de facto* NspmSyns if the substrate Nspd is present and if Spd does not completely outcompete Nspd as substrate. This is of direct relevance in some bacteria, for example, Nspm is a prominent polyamine in *Thermodesulfobacterium* *thermophilum* and in *Thermus thermophilus* (30), each of which encodes a TspmSyn. The NspmSyn activity of a crenarchaeote TspmSyn has also been demonstrated (46), and in the case of *Pyrobaculum* *calidifontis*, the Nspd substrate for Nspm biosynthesis is thought to be generated from Tspm by an unknown dehydrogenase. TspmSyns from *Arthrospira* *platensis,* *Fimbriimonas* *ginsengisoli*, and *Thermodesulfobacterium* *thermophilum*, and the Spm/Tspm synthases from *Ca. Pelagibacter* species were also able to produce Nspd from Dap, revealing that TspmSyns are also potential Nspd and Nspm synthases, if Dap or Nspd are present in the corresponding cells. Although all SpmSyns aminopropylated Hspd, only the SpmSyn from *Oceanithermus* *profundus* was able to aminopropylate Nspd, suggesting that most SpmSyns exhibit strict specificity for aminopropylating substrates with *N*-aminobutyl groups (Put, Spd, and Hspd).

One of our more unexpected findings was that most of the SpdSyns we analyzed were able to aminopropylate Agm. Until our study, only the APTs of *Thermus thermophilus* and the archaea *Thermococcus* *kodakarensis* and *Pyrobaculum* *calidifontis* were known to aminopropylate Agm (33, 42, 46). Some of the SpdSyns were able to aminopropylate Agm in the *E. coli* BL21*speG* strain that contains a very high level of putrescine and relatively low level of Agm. The overexpressed *E. coli* SpdSyn was only able to produce *N*^1^-APAgm in the BL21*speB* strain that contains a very high level of Agm and a relatively low level of Put. When expressed in BL21*speB* grown with L-arginine, the only bacterial SpdSyn that was not able to aminopropylate Agm was the SpdSyn (SpeE) of *P*. *aeruginosa* PAO1. It is notable that *E. coli* and *P*. *aeruginosa* PAO1 are the only species we examined that encode two alternative pathways for Put biosynthesis *via* L-arginine and L-ornithine decarboxylases. Most of the species encoding *N*^1^-APAgm synthases possess only L-arginine decarboxylases, although these nonhomologous decarboxylases are derived from four different protein folds. Therefore, irrespective of the evolutionary origin of agmatine production within these species, *N*^1^-APAgm biosynthesis, and potentially the formation of Spd *via N*^1^-APAgm is likely a common feature of bacterial polyamine biosynthesis. This seems to be the case with even well-studied species such as *Bacillus subtilis*, which is known to accumulate Spd without any detectable Put accumulation (47). It is also the case that *Oceanithermus* *profundus*, *Thermosynthropha lipolytica*, *Geobacillus* *stearothermophilus,* *Sulfobacillus* *acidophilus*, and *Desulfosporosinus* *orientis* contain little or no detectable Put accumulation (30). *N*^1^-APAgm may therefore play a much more widespread role in Spd biosynthesis than previously realized, and the biosynthetic sequence of Spd biosynthesis from L-arginine will need to be reconfigured for many bacteria.

We also note that some APT pairs where the SpdSyn produces *N*^1^-APAgm, when coexpressed with their corresponding TspmSyn, are able to aminopropylate *N*^1^-APAgm, that is, to produce *N*^12^-guanidinothermospermine. It is therefore formally possible that in some species, Tspm could be produced from Agm *via N*^1^-APAgm and *N*^12^-guanidinothermospermine, in the absence of Put and Spd formation. Whether the same ureohydrolase would act on both *N*^1^-APAgm and *N*^12^-guanidinothermospermine is not known but the advantage of such a pathway would be that Tspm biosynthesis could be decoupled from both Put and Spd biosynthesis. It is likely that the various *N*^1^-APAgm synthases identified in our study were able to produce Spd directly from Put in *E. coli* BL21*speE* because the *E. coli* agmatine ureohydrolase rapidly converts Agm to Put. Thus, Put would be the only substrate available for aminopropylation.

To distinguish between Spm and Tspm synthases at the amino acid sequence level, the most useful motif is hhhhGGGD(G/C)G for SpmSyns and hhhhGGGEGA for TspmSyns, where h represents a small hydrophobic residue. The exceptions are the TspmSyns from *Thermodesulfobacterium* *thermophilum*, and *Fimbriimonas* *ginsengisoli*, which have a SpmSyn-like motif. This motif is part of the dcAdoMet binding region (8). The motif does not distinguish SpdSyns/*N*^1^-APAgmSyns from either Spm or Tspm synthases. A Maximum Likelihood phylogenetic tree confirms the separation of Spm and Tsm synthases into two distinct clades but noticeably each clade contains SpdSyns/*N*^1^-APAgmSyns.

The most unexpected bacterial species from which we functionally identified a TspmSyn is the important opportunistic human pathogen *P*. *aeruginosa*. Indeed, the role of polyamines in the growth, virulence and biofilm formation of *P. aeruginosa* PAO1 and PA14 strains have been extensively studied (48, 49, 50, 51). The TspmSyn that we identified corresponds to the PAO1 strain PA4774 gene, also known as *speE2*, which encodes the protein NP_253462 (349 a.a.). This gene has been previously described as encoding a SpdSyn (52, 53) and a NspdSyn (40). *P. aeruginosa* is unusual in that it encodes a typical γ-proteobacterial class 1a AdoMetDC (SpeD) and SpdSyn (SpeE, NP_250378, 286 aa), which are not physically linked in the PAO1 genome. It also encodes a smaller class 1b AdoMetDC (SpeD2, PA7443) immediately upstream of the atypical APT, SpeE2 (the TspmSyn).

Biochemical evidence for SpeE2 being a SpdSyn is based on changes to surface-associated Spd levels when the *speE2* gene is mutated (53). This evidence is rather marginal as the reduction of Spd level is only 2-fold. The evidence for SpeE2 being a NspdSyn, or at least being required for Nspd biosynthesis is more compelling; mutation of *speE2* was found to reduce surface-associated Nspd by 800-fold (40). We have shown that *P. aeruginosa* SpeE2 (TspmSyn) does not convert Dap to Nspd. Nevertheless, it is formally possible that the TspmSyn might be required for Nspd production. In the lycophyte vascular plant *Selaginella* *lepidophylla*, Tspm is oxidized to Nspd by a polyamine oxidase SelPAO5 (41). The *P. aeruginosa* PAO1 spermidine dehydrogenase SpdH is able to catabolize Spd to Dap and 4-aminobutyraldehyde, and Spm to Spd and 3-aminopropanaldehyde (54, 55). Although Tspm was not tested as a substrate, it seems plausible that Tspm could be catabolized to Nspd and 4-aminobutyraldehyde. Thus, it is possible that the Tspm could be catabolized to Nspd by SpdH, however, we were able to show that the TspmSyn efficiently converts Nspd to Nspm. A futile cycle might then be set up where Spd is converted to Tspm, which is catabolized to Nspd, which is then converted to Nspm, and then catabolized back to Nspd.

Our analysis of the PAO1 Spd dehydrogenase amino acid sequence (NP_252402; 620 aa) by Interproscan shows that the N-terminal region encodes a strong twin arginine translocation pathway sequence. This would suggest that the Spd dehydrogenase SpdH is exported fully folded into the periplasm. A periplasmic location of SpdH would prevent Nspd being convert to Nspm by the TspmSyn, and would facilitate the export of Nspd to the cell surface in response to cationic antibiotics. This hypothetical pathway for Nspd biosynthesis offers an alternative to the characterized Nspd biosynthetic pathway *via* Dap and carboxyNspd (56). The *P. aeruginosa* SpeE SpdSyn exhibits 60% amino acid identity to the *E. coli* SpdSyn, but the SpeE2 TspmSyn exhibits only 36% identity to *E. coli* SpdSyn, suggesting that the *P. aeruginosa* SpeD2-SpeE2 Tspm biosynthetic metabolon was acquired from outside the Proteobacteria by horizontal gene transfer.

## Experimental procedures

### Bacterial strains and growth conditions

All strains used are derived from *E. coli* BL21 (DE3). Construction of BL21*speD* (*S*-adenosylmethionine decarboxylase gene deletion), BL21*speE* (spermidine synthase gene deletion), and BL21*speG* (spermidine *N*-acetyltransferase gene deletion) was described previously (39, 57, 58). A new strain BL21*speB* (BL21 (DE3) Δ*speB*::FRT-*kan*^+^-FRT; agmatine ureohydrolase gene deletion) was constructed for the current study. The Δ*speB*::FRT-*kan*^+^-FRT locus derived from strain JW2904 of the KEIO collection was transduced into BL21 (DE3) using phage P1 and selected for using kanamycin resistance. Flux from L-glutamate to L-ornithine was inhibited by growing BL21*speB* with added L-arginine as indicated. All strains were grown on or in polyamine-free, chemically defined M9 medium (59).

### Chemicals

Thermospermine was purchased from Santa Cruz Biotechnology Inc. Homospermidine was a kind gift from Patrick Woster, Medical University of South Carolina. All other chemicals were purchased from Sigma Aldrich.

### Plasmids, genes, and expression in *E. coli* BL21 (DE3)

All tested genes were synthesized by GenScript with *E. coli*-optimized codons and inserted into pETDuet-1 or pACYCDuet-1 (Novogen) with 5′-Nde1 and 3′-Xho1 sites. Genes were expressed from a phage T7 promoter in pETDuet-1 or from pACYCDuet-1 as indicated and selected with ampicillin or chloramphenicol, respectively. GenBank protein accession numbers and protein sizes in amino acids of each APT homolog are listed in Table S1, along with preferred growth mode and percentage amino acid identity of pairs of APTs encoded by the same genome.

### Bacterial growth and polyamine extraction

Strains derived from *E. coli* BL21 were grown in M9 polyamine-free chemically defined medium without or with added polyamines as described previously (3). Polyamine extraction and benzoylation was performed as described previously (3).

### Liquid chromatography-mass spectrometry

Benzoylated cell extract samples were run on an Agilent 1290 Infinity HPLC system fitted with an Eclipse XDB-C18 column (4.6 × 150 mm, 5 μm particle size), coupled to an Agilent 6130 quadrapole ESI mass spectrometer run in positive mode, employing a scan range of 100 to 1100 m/z. Due to the relatively low resolution of this machine, the mass tolerance window used for the EICs was set to 1.0 Da. For the liquid chromatography stage, a flow rate of 0.5 ml/min at 20 °C was used with a 5 μl injection volume, employing a gradient elution with aqueous acetonitrile containing 0.1% formic acid. The different polarities of the analyzed compounds required gradient adjustment.

### LC-MS/MS analysis of tetrabenzoylated Spm and Tspm

High performance liquid chromatography conditions are as follows: reverse phase chromatography was performed using an ACE 3 C18-PFP 150 × 4.6 mm, 3 μm HPLC column (Mac-Mod). Column temperature, sample injection volume, and flow rate was set to 30 °C, 5 μl, and 0.8 ml/min, respectively. HPLC conditions were as follows: solvent A: water with 0.1% formic acid (v/v), Optima LC/MS Grade; solvent B: acetonitrile with 0.1% formic Acid (v/v), Optima LC/MS Grade: 40% B, 0 to 13 min; 5% B, 15 to 18 min; 95% B, 20 to 23 min; 40% B, 24 to 30 min. Total run time 30 min. Data was processed by SCIEX MultiQuant 3.0.3 software (https://sciex.com/products/software/mass-spectrometry-software-solutions) (AB Sciex) with relative quantification based on the peak area of each metabolite. Targeted mass spectrometric analyses were performed on an AB Sciex QTRAP 6500+ mass spectrometer equipped with an ESI ion spray source. The ESI source was used in positive ion mode. Ion source conditions in the positive mode were as follows: ion source gas 1, 70 p.s.i.; ion source gas 2, 65 p.s.i.; curtain gas, 45 p.s.i.; ion spray voltage, 5500 V; and source temperature, 550 °C. Data acquisition was performed in MRM mode. Three diagnostic MRM transitions in the positive mode for tetrabenzoylated spermine (elution at 11.50 min) and thermospermine (10.63 min) were obtained, and three MS ion transitions Q1/Q3, 619.228/497.2; 619.228/162 and 619.228/77 were monitored. The MS transition of Q1/Q3, 619.228/497.2, was used as the quantifier ion while 619.228/162 and 619.228/77 were used as the qualifier ions. The mass spectrometer was coupled to a Shimadzu HPLC (Nexera X2 LC-30AD) and was controlled by Analyst 1.7 software (https://sciex.com/products/software/mass-spectrometry-software-solutions).

### LC-HRMS analysis of tetrabenzoylated N^1^-aminopropylagmatine and pentabenzoylated N^12^-guanidinothermospermine

For the chromatographic analysis of tetrabenzoylated *N*^1^-aminopropylagmatine only (Fig. S10), Kinetex 2.6 μm, C18, 100 A 50 × 2.1 mm column (Phenomenex) was used with a total run time of 7.5 min. To analyze tetrabenzoylated *N*^1^-aminopropylagmatine and pentabenzoylated *N*^12^-guanidinothermospermine together, an ACE 3 C18-PFP 150 × 4.6 mm HPLC column (Mac-Mod, USA) with total run 30 min was used to obtain a sharper and more symmetrical peak for pentabenzoylated *N*^12^-guanidinothermospermine. For HPLC using the ACE 3 C18-PFP 150 × 4.6 mm column, column temperature, sample injection volume, the flow rate was set to 30 °C, 5 μl, and 0.5 ml/min, respectively. HPLC conditions were as follows: solvent A: water with 0.1% formic Acid (v/v), Optima LC/MS Grade. Solvent B: acetonitrile with 0.1% Formic Acid (v/v), Optima LC/MS Grade: 2% B, 0 to 2 min; 90% B, 5 to 16 min; 2% B, 17 to 30 min. Total run time: 30 min. Data was processed by SCIEX OS software version 2.0.1.48692 (AB Sciex) with relative quantification based on the peak area of each metabolite.

Untargeted mass spectrometric analyses were performed on a Sciex TripleTOF 6600 system (AB SCIEX) equipped with an electrospray ionization source used in the positive ionization mode and configured as follows: ion source gas 1, 50 p.s.i; ion source gas 2, 45 p.s.i; curtain gas, 25 p.s.i.; source temperature, 550 °C; and ion spray voltage floating, +5500 V. TOF-MS mode (full scan) and information dependent acquisition mode (product ion scan) were utilized to collect MS and MS/MS data, respectively. For TOF-MS scans, the mass range was from m/z 60 to 1000, and for product ion scans, the mass range was from m/z 30 to 1000. The collision energy was set at 30 V (+) and collision energy spread was ±15 V. Accumulation time was 0.25 s for TOF-MS scans and 0.06 s for product ion scans. The instrument was automatically calibrated using a calibration delivery system injected in APCI positive calibration solution every 5 samples. The mass spectrometer was coupled to a Shimadzu HPLC (Nexera X2 LC-30AD), and system was controlled by Analyst TF 1.8.1 software (https://sciex.com/products/software/mass-spectrometry-software-solutions) (Sciex).

### Phylogenetic analysis

Aminopropyltransferase homologs were identified in specific bacterial genomes using BLASTP analysis, employing the SpdSyn amino acid sequences from *E. coli* and *Bacillus subtilis* and the *A. thaliana* Tspm synthase encoded by the *acl5* gene (Genbank protein accession numbers listed in Table S1). A general identification of genomes encoding two APT homologs was performed by interrogating all bacterial genomes using TBLASTN. The N-terminal regions of the *H. sapiens* SpmSyn and the *Ca. Pelagibacter* species Spd/Spm/Tspm synthases were removed to facilitate alignment. Amino acid sequence alignment was performed with ClustalW for Figure 9 or MUSCLE (60) for phylogenetic tree creation, and Maximum Likelihood phylogenetic tree construction was performed with IQ-TREE (61), using the “Auto” substitution model and 1000 ultrafast bootstraps analysis (62). The Maximum Likelihood phylogenetic tree was visualized with iTOL (63).

## Data availability

All data presented are contained within the article.

## Supporting information

This article contains supporting information.

## Conflict of interest

The authors declare that they have no conflicts of interests with the contents of this article.

## Supporting information

Supporting Informtion
