# Supplementary material for: Functional identification of bacterial spermine, thermospermine, norspermine, norspermidine, spermidine, and N1-aminopropylagmatine synthases
Source: J Biol Chem. 2024 Apr 6;300(5):107281. doi: 10.1016/j.jbc.2024.107281 (PMC11107197; doi:10.1016/j.jbc.2024.107281)
Supplement: Supporting Informtion [file mmc1.pdf]

Supporting Information

Functional identification of bacterial spermine, thermospermine, norspermine, norspermidine, spermidine, and N^1-aminopropylagmatine synthases

Bin Li[1], Jue Liang[1], Hamid R. Baniasadi[1], Shin Kurihara[2], Margaret A. Phillips[1] and Anthony J. Michael[1*]

[1]Department of Biochemistry, UT Southwestern Medical Center, Dallas, Texas, USA

[2]Faculty of Biology-Oriented Science and Technology, Kindai University, Kinokawa, Wakayama, Japan

[*]For correspondence: Anthony J. Michael, anthony.michael@utsouthwestern.edu

Table S1. Aminopropyltransferase homologs analyzed in this study.

Species (Phylum, Class)	Protein [GenBank Acc. No.]	Size (a.a.)	Growth mode	Pairwise a.a. % Identity
Arabidopsis thaliana	**SpdSyn** [Q9ZUB3]	334	n/a	
(Viridiplantae, Streptophyta)	**SpmSyn** [BAH19534]	359		
	**TspmSyn** [OAO96167]	339		
Arthrospira platensis NIES-39	**APT01** [BAI92260]	313	M	60
(Cyanobacteriota)	**APT02** [BAI90257]	313		
Bacillus subtilis 168	**SpdSyn** [NP_391630]	276	M	
(Bacillota, Bacilli)				
Chlamydomonas reinhardtii	**SpdSyn** [XP_001702843]	318	n/a	35
(Viridiplantae, Chlorophyta)	**TspmSyn** [ADF43120]	314		
Desulfarculus baarsii	**APT01** [WP_013259899]	304	M	34
(Thermodesulfobacteriota)	**APT02** [WP_013257411]	309		
Desulfosporosinus orientis	**APT01** [WP_014182696]	284	M	41
(Bacillota, Clostridia)	**APT02** [WP_014182917]	291		
Dictyoglomus thermophilum	**APT01** [WP_012546901]	304	T	42
(Dictyoglomota)	**APT02** [WP_012547113]	285		
Escherichia coli	**SpdSyn** [KIG32097]	299	M	
(Pseudomonadota, γ-Proteobacteria)				
Fimbriimonas ginsengisoli	**APT01** [WP_025227485]	279	M	33
(Armatimonadota)	**APT02** [WP_025225227]	308		
Geobacillus stearothermophilus	**APT01** [WP_049624640]	275	T	43
(Bacillota, Bacilli)	**APT02** [WP_033014118]	297		
Heliorestis convoluta	**APT01** [WP_153726121]	279	M	48
(Bacillota, Clostridia)	**APT02** [WP_153723875]	288		
Homo sapiens	**SpdSyn** [[NP_003123]	302	n/a	29
(Metazoa, mammalia)	**SpmSyn** [CAA88921]	368		
Leptospirillum ferrodiazotrophum	**APT** [EES53973]	306	M	
(Nitrospirota)				
Microcystis aeruginosa (Cyanobacteriota)	**APT** [WP_002790803]	315	M	
Oceanithermus profundus (Deinococcota)	**APT** [WP_013456826]	290	T	
Ca. Pelagibacter sp. HTCC7211	**APT** [WP_008544956]	346	M	
Ca. Pelagibacter ubique HTCC1062	**APT** [WP_011282223]	346	M	
(Pseudomonadota, α-Proteobacteria)				
Pseudomonas aeruginosa PAO1	**APT01** [NP_250378]	286	M	38
(Pseudomonadota, γ-Proteobacteria)	**APT02** [NP_253462]	349		
Rhodothermus marinus	**APT01** [WP_012844841]	307	T	39
(Rhodothermota)	**APT02** [WP_012844658]	296		
Sulfobacillus acidophilus TPY	**APT01** [AEJ41924]	275	T	38
(Bacillota, Clostridia)	**APT02** [AEJ39917]	288		
Thermoanaerobacter brockii	**APT** [WP_003867984]	277	T	
(Bacillota, Clostridia)				
Thermobrachium celere (Bacillota, Clostridia)	**APT** [CDF58371]	280	T	
Thermodesulfobacterium thermophilum	**APT01** [WP_022855228]	273	T	34
(Thermodesulfobacteriota)	**APT02** [WP_022855378]	302		
Thermodesulfovibrio yellowstonii	**APT01** [ACI21680]	278	T	37
(Nitrospirota)	**APT02** [ACI21563]	299		
Thermosyntropha lipolytica	**APT01** [SHG87240]	277	T	50
(Bacillota, Clostridia)	**APT02** [SHG61007]	275		
Thermotoga maritima MSB8	**APT** [AGL49579]	293	T	
(Thermotogota)				
Thermus thermophilus (Deinococcota)	**APT** [WP_011172918]	314	T	
**Uncultured Caudovirales phage**	**APT** [CAB4129971]	280	M	

Phyla are presented in parentheses. APT01 and APT02 represent pairs of aminopropyltransferase homologs encoded by the same genome. APT represents singleton aminopropyltransferase homologs. GenBank protein accession numbers in brackets. T, thermophile; M, mesophile, n/a, not applicable (eukaryotes). Pairwise amino acid identities are given for pairs of homologs encoded by the same genome.

Table S2. Presence of L-arginine decarboxylase and L-ornithine decarboxylase homologs in genomes used in this study.

Species (Phylum)	Protein [acc.no.] size a.a.	fold
Arthrospira platensis (Cyanobacteriota)	[WP_014276307] 653	AR
Bacillus subtilis (Bacillota, Bacilli)	[WP_003244780] 490	AAT
Desulfarculus baarsii (Thermodesulfobacteriota)	[WP_013256978] 190	pyruvoyl
Desulfosporosinus orientis (Bacillota, Clostridia)	[WP_014182695] 483	AAT
Dictyoglomus thermophilum (Dictyoglomota)	[WP_012547791] 133	AdoMetDC
Escherichia coli (Pseudomonadota, γ-Proteobacteria)	ADC [WP_101348650] 658	AR
	ODC [BAE77028] 711	AAT
Fimbriimonas ginsengisoli (Armatimonadota)	[MBI3721038] 127	AdoMetDC
Geobacillus stearothermophilus (Bacillota, Bacilli)	[WP_047818078] 490	AAT
Heliorestis convoluta (Bacillota, Clostridia)	[WP_153725798] 497	AAT
Leptospirillum ferrodiazotrophum (Nitrospirota)	[EES53974] 485	AAT
Microcystis aeruginosa (Cyanobacteriota)	[WP_158199734] 679	AR
Oceanithermus profundus (Deinococcota)	N.D.	
Pseudomonas aeruginosa PAO1(γ-Proteobacteria)	ADC [NP_253526] 636	AR
	ODC [WP_003094617] 387	AR
Rhodothermus marinus (Rhodothermota)	[WP_012843912] 653	AR
Sulfobacillus acidophilus (Bacillota, Clostridia)	[AEJ41925] 153	pyruvoyl
Thermoanaerobacter brockii (Bacillota, Clostridia)	[WP_012269266] 495	AAT
Thermobrachium celere (Bacillota, Clostridia)	[WP_018661112] 487	AAT
Thermodesu. thermophilum (Thermodesulfobacteriota)	[WP_022854912] 148	AdoMetDC
Thermodesulfovibrio yellowstonii (Nitrospirota)	ODC [WP_012545474] 372	AR
Thermosyntropha lipolytica (Bacillota, Clostridia)	[WP_073092403] 499	AAT
Thermotoga maritima (Thermotogota)	ODC [WP_004082424] 388	AR
Thermus thermophilus (Deinococcota)	[WP_011173677] 630	AR

Proteins are L-arginine decarboxylase (ADC) homologs unless noted. AR, alanine racemase fold; AAT, aspartate aminotransferase fold; pyruvoyl, pyruvoyl-dependent L-arginine decarboxylase similar to L-arginine decarboxylase of *Methanocaldococcus jannaschii;* AdoMetDC, similar to *S*-adenosylmethionine decarboxylase: ODC, L-ornithine decarboxylase; N.D., not detected.

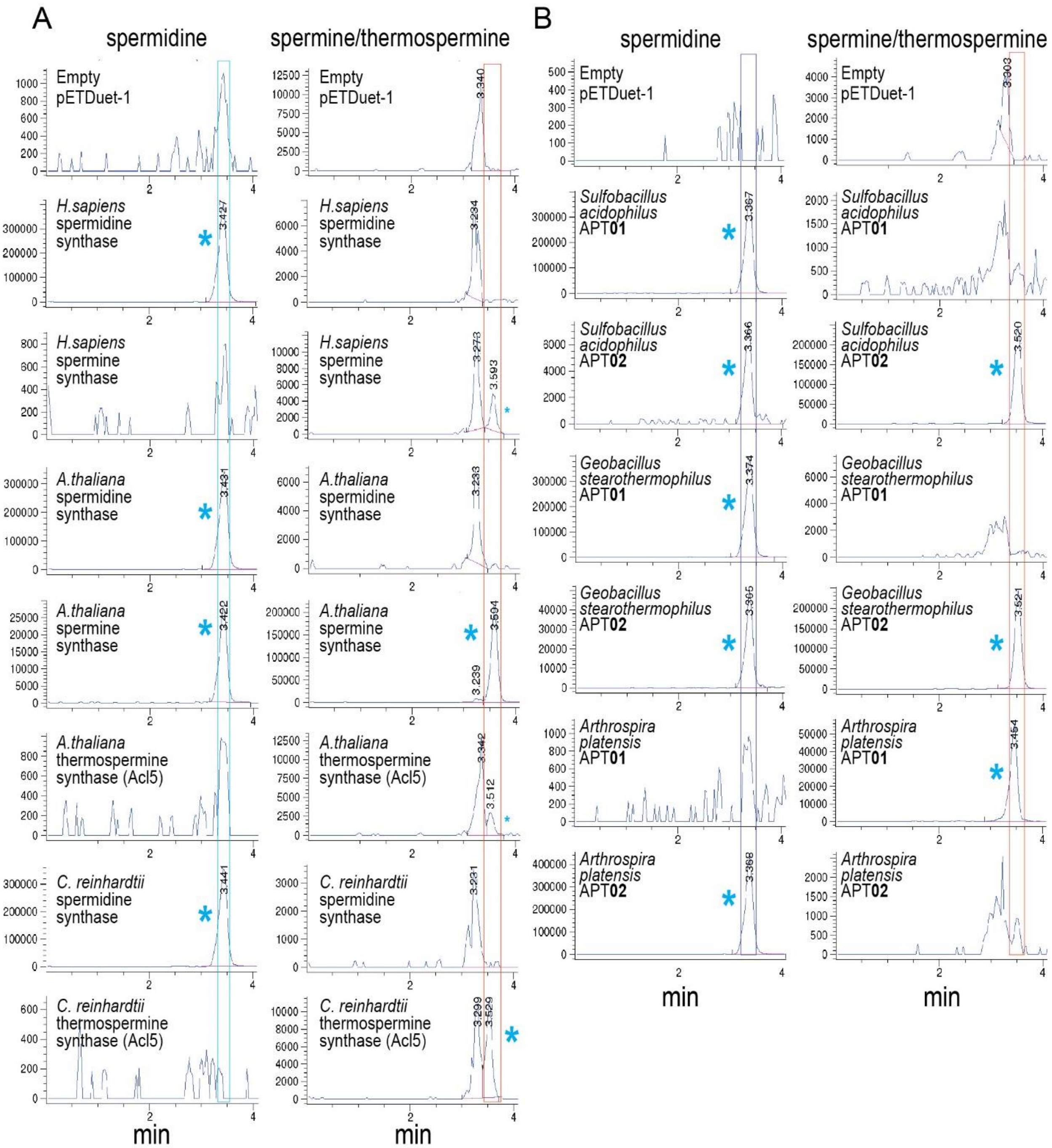

Figure S1. Expression of aminopropyltransferases in spermidine-devoid *E. coli* BL21*speE*. *A* and *B* are independent experiments. Within *A* or *B*, cultures were grown and processed in parallel. Shown are the LC-MS Extracted Ion Chromatograms for tribenzoylated spermidine (Spd, EIC, mass tolerance window = 457.94:458.94, blue box) and tetrabenzoylated spermine/thermospermine (Spm/Tspm, EIC = 619.02:620.02, red box). The presence of peaks for Spd or Spm/Tspm are highlighted by blue asterisks. All genes were expressed from pETDuet-1.

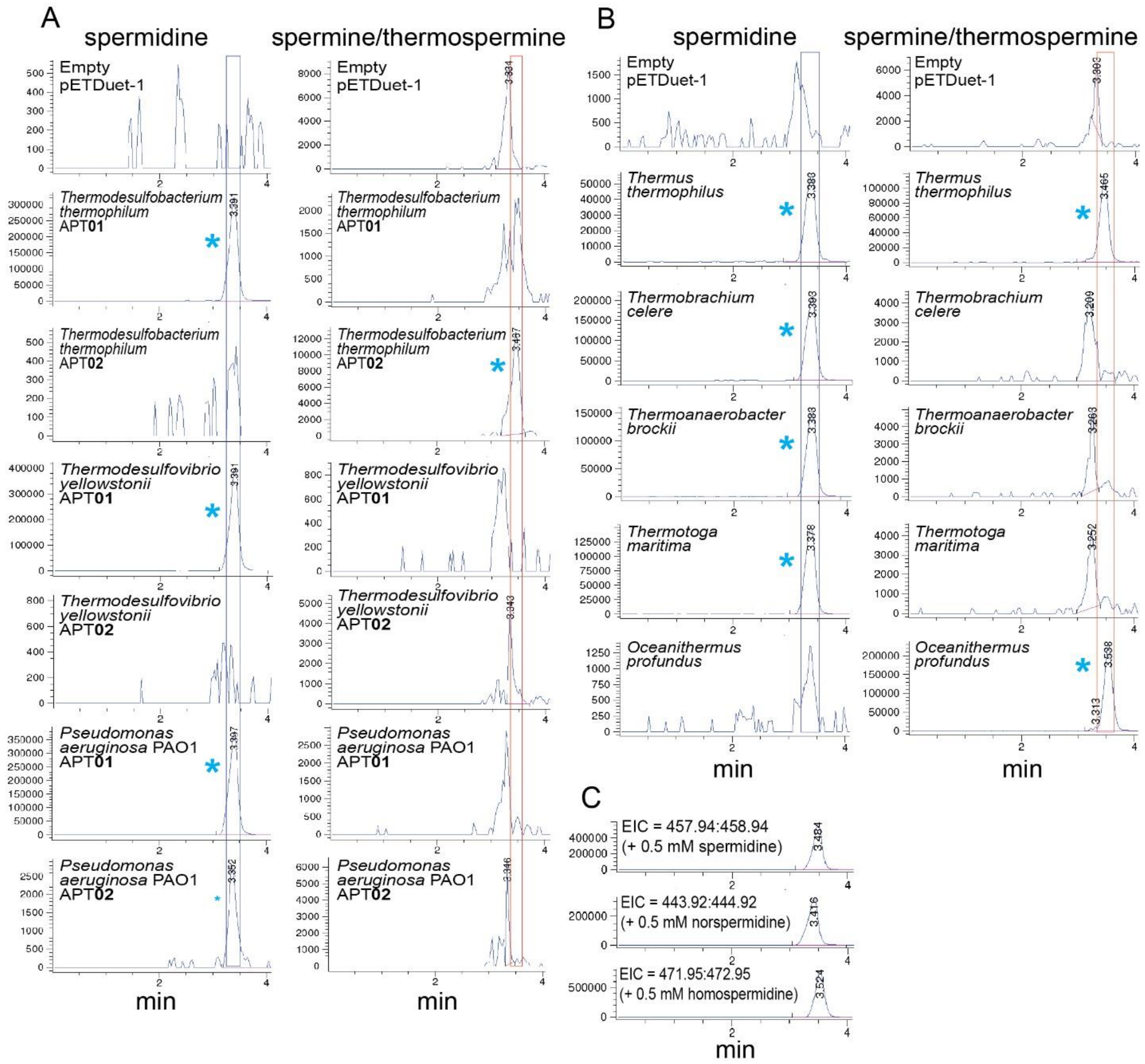

Figure S2. Expression of aminopropyltransferases in spermidine-devoid *E. coli* BL21*speE*. *A* and *B* are independent experiments. Within *A* or *B*, cultures were grown and processed in parallel. Shown are the LC-MS Extracted Ion Chromatograms for tribenzoylated spermidine (Spd, EIC = 457.94:458.94, blue box) and tetrabenzoylated spermine/thermospermine (Spm/Tspm, EIC = 619.02:620.02, red box). The presence of peaks for Spd or Spm/Tspm are highlighted by blue asterisks. *C*, *E. coli* spermidine-devoid BL21*speE* strain with empty pETDuet-1, grown in M9 medium with either 0.5 mM pure spermidine, norspermidine or homospermidine. EICs for tribenzoylated spermidine (457.94:458.94), norspermidine (443.93:444.92) and homospermidine (471.95:472.95) show corresponding peaks. Different ion intensities may relate to different efficiencies of uptake or *N*-acetylation, or both. All genes were expressed from pETDuet-1.

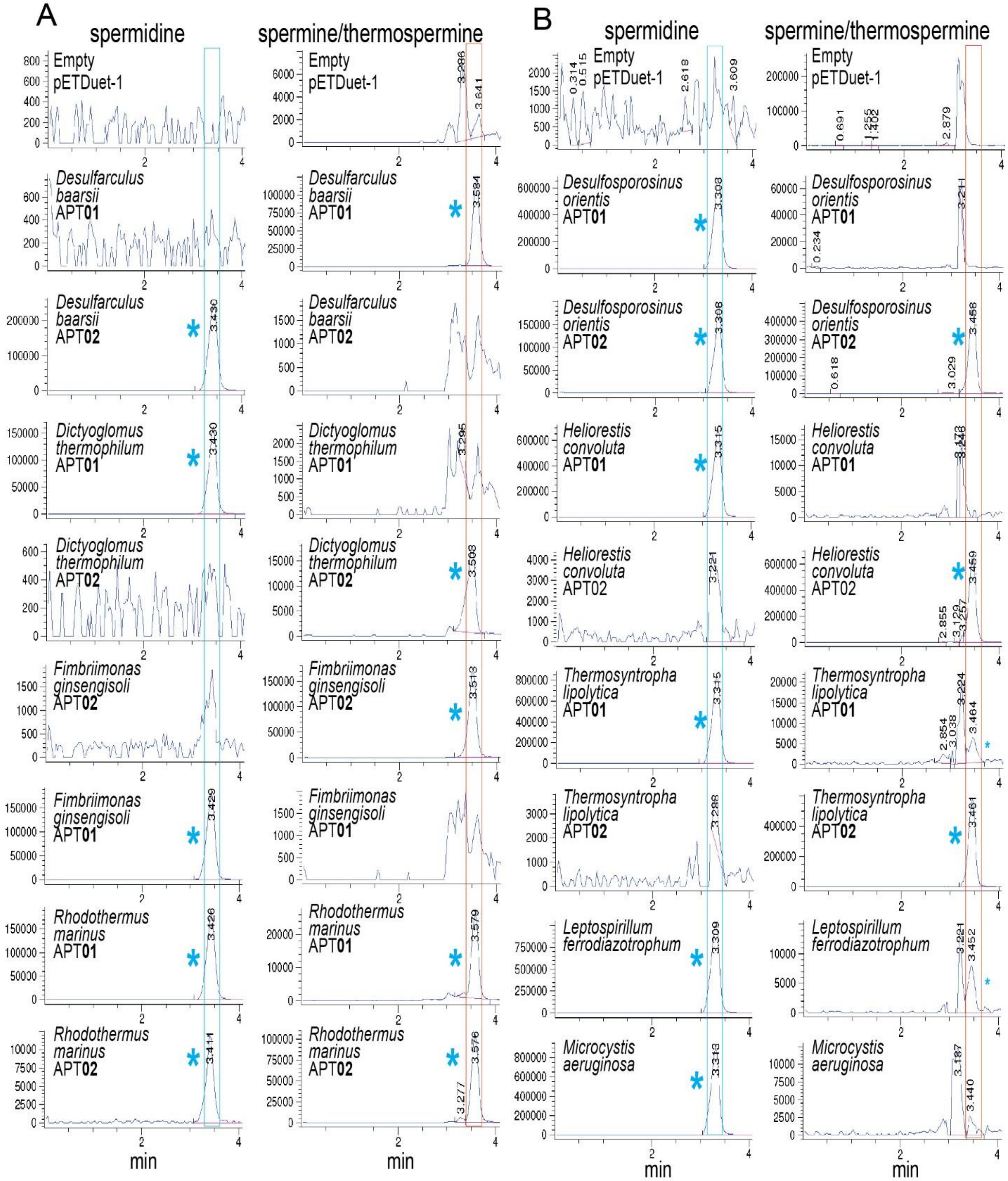

Figure S3. Expression of aminopropyltransferases in spermidine-devoid *E. coli* BL21*speE*. *A* and *B* are independent experiments. Within *A* or *B*, cultures were grown and processed in parallel. Shown are the LC-MS Extracted Ion Chromatograms for tribenzoylated spermidine (Spd, EIC = 457.94:458.94, blue box) and tetrabenzoylated spermine/thermospermine (Spm/Tspm, EIC = 619.02:620.02, red box). The presence of peaks for Spd or Spm/Tspm are highlighted by blue asterisks. All genes were expressed from pETDuet-1.

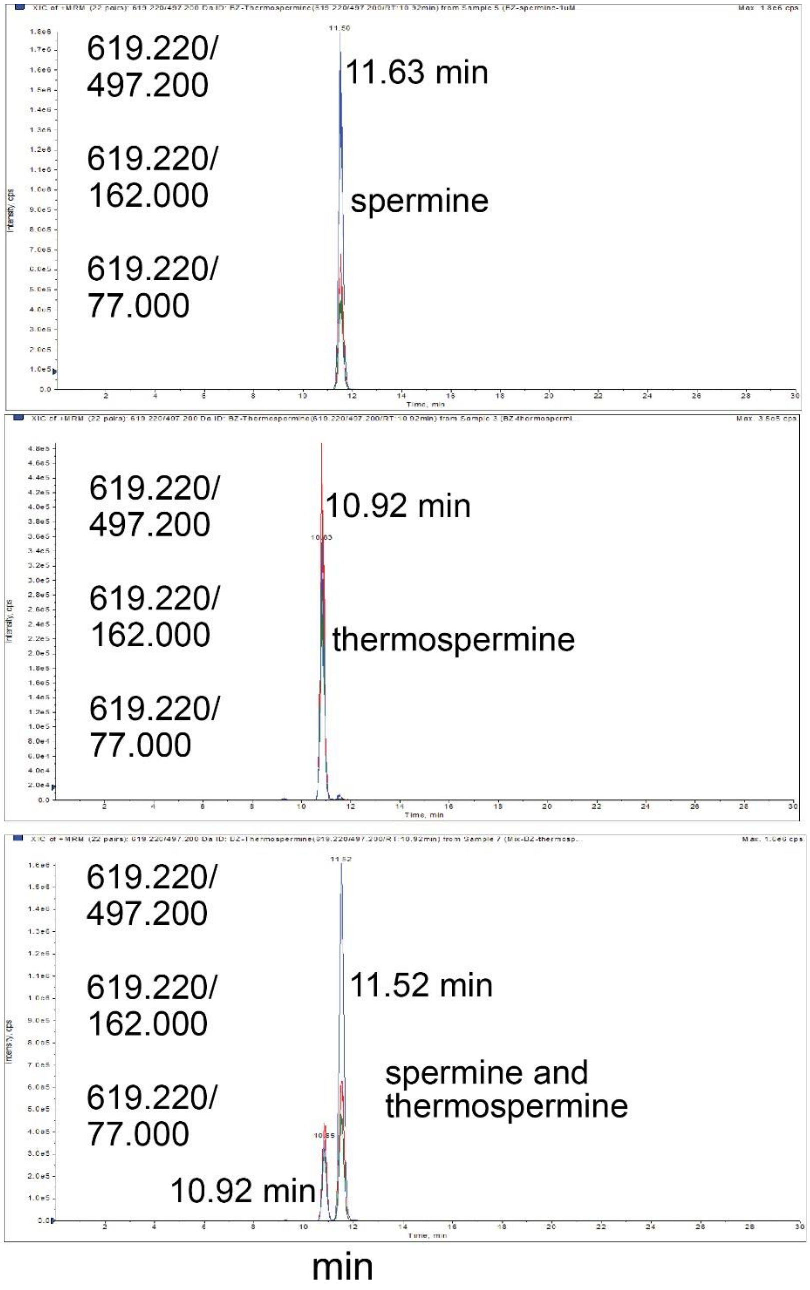

Figure S4. LC-MS/MS chromatographic separation of benzoylated spermine and thermospermine chemical standards. Shown are overlapping peaks for Extracted Ion Chromatograms of tetrabenzoylated spermine and thermospermine recoded in MRM positive polarity mode showing three MRM transitions 619.228/497.2, 619.228/162, and 619.228/77. The ion 619.228/497.2 was used as the quantifier ion while 619.228/162, and 619.228/77 were used as the qualifier ions. Bottom panel shows a mixture of an equal amount of spermine and thermospermine. The differences in relative quantities of tetrabenzoylated spermine and thermospermine is due to the commercially obtained thermospermine containing a large amount of norspermidine.

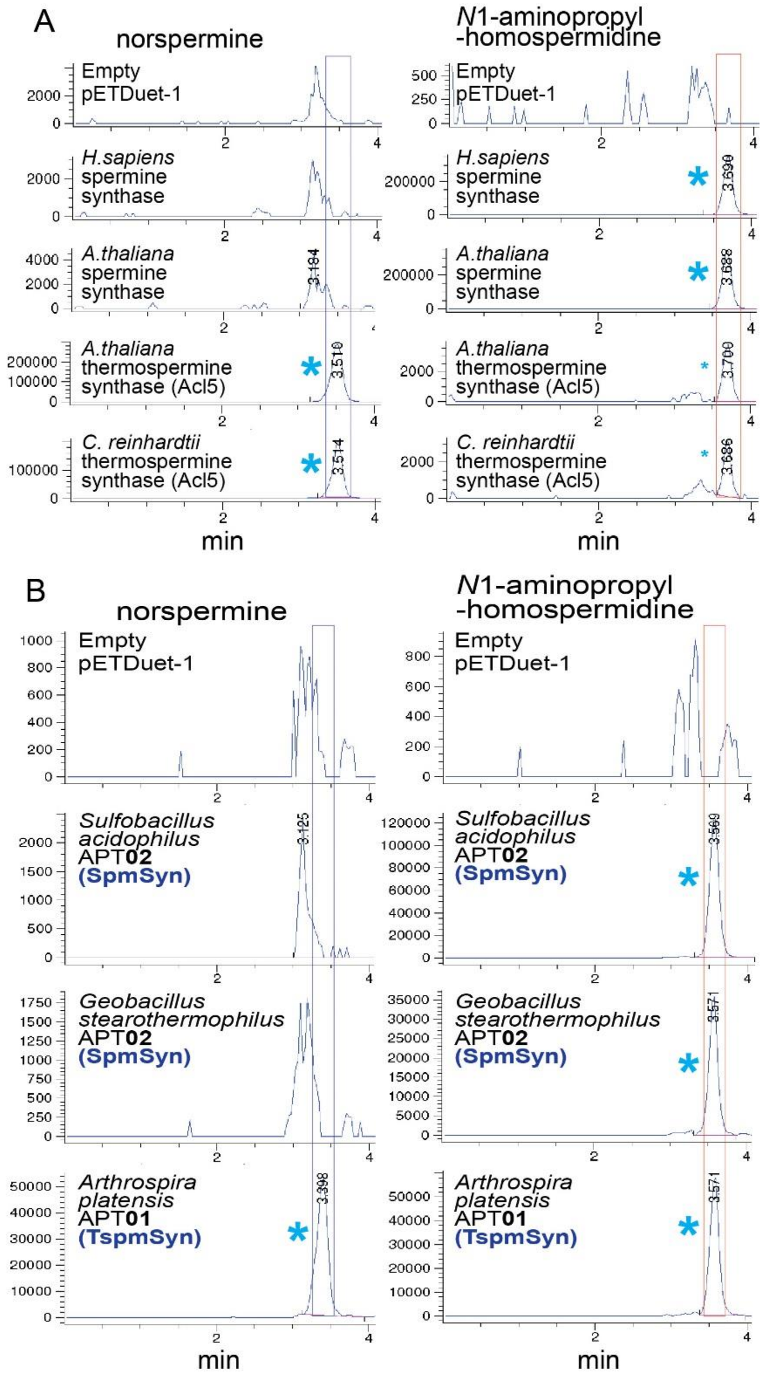

Figure S5. Expression of aminopropyltransferases in spermidine-devoid *E. coli* BL21*speE* grown with 0.5 mM norspermidine or homospermidine. *A* and *B* are independent experiments. Left panel samples were grown with 0.5 mM norspermidine, right panel with 0.5 mM homospermidine in M9 medium. Within *A* or *B*, cultures were grown and processed in parallel. Shown are the LC-MS Extracted Ion Chromatograms for tetrabenzoylated norspermine (Nspd, EIC = 605:606, blue box) and tetrabenzoylated N^1-aminopropylhomospermidine (N^1-APHspd, EIC = 619.02:620.02, red box). The presence of peaks for Nspd or N^1-APHspd are highlighted by blue asterisks. SpmSyn, spermine synthase; TspmSyn, thermospermine synthase. All genes were expressed from pETDuet-1.

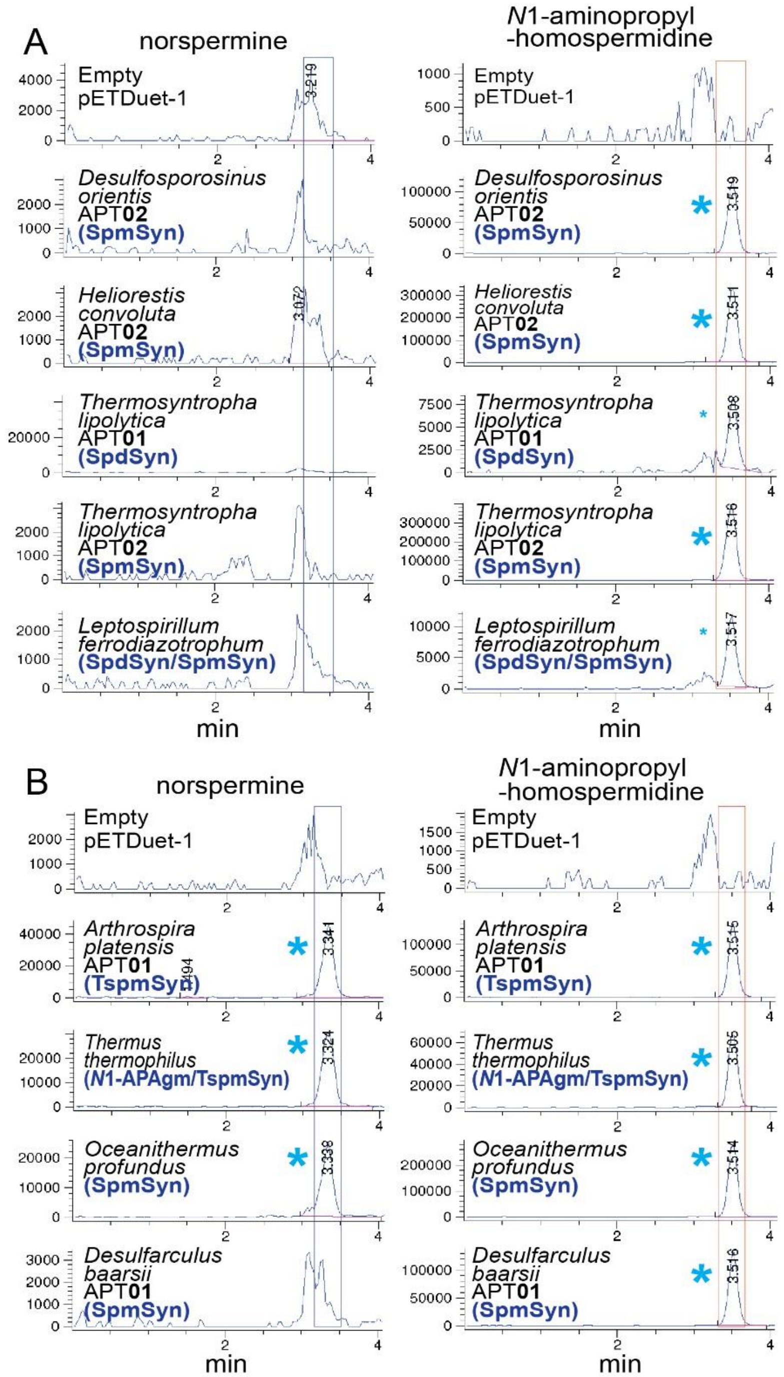

Figure S6. Expression of aminopropyltransferases in spermidine-devoid *E. coli* BL21*speE* grown with 0.5 mM norspermidine or homospermidine. *A* and *B* are independent experiments. Left panel samples were grown with 0.5 mM norspermidine, right panel with 0.5 mM homospermidine in M9 medium. Within *A* or *B*, cultures were grown and processed in parallel. Shown are the LC-MS Extracted Ion Chromatograms for tetrabenzoylated norspermine (Nspd, EIC = 605:606, blue box) and tetrabenzoylated N^1-aminopropylhomospermidine (N^1-APHspd, EIC = 619.02:620.02, red box). The presence of peaks for Nspd or N^1-APHspd are highlighted by blue asterisks. SpmSyn, spermine synthase; TspmSyn, thermospermine synthase; SpdSyn, spermidine synthase; *N*1-APAgm, N^1-aminopropylagmatine. All genes were expressed from pETDuet-1.

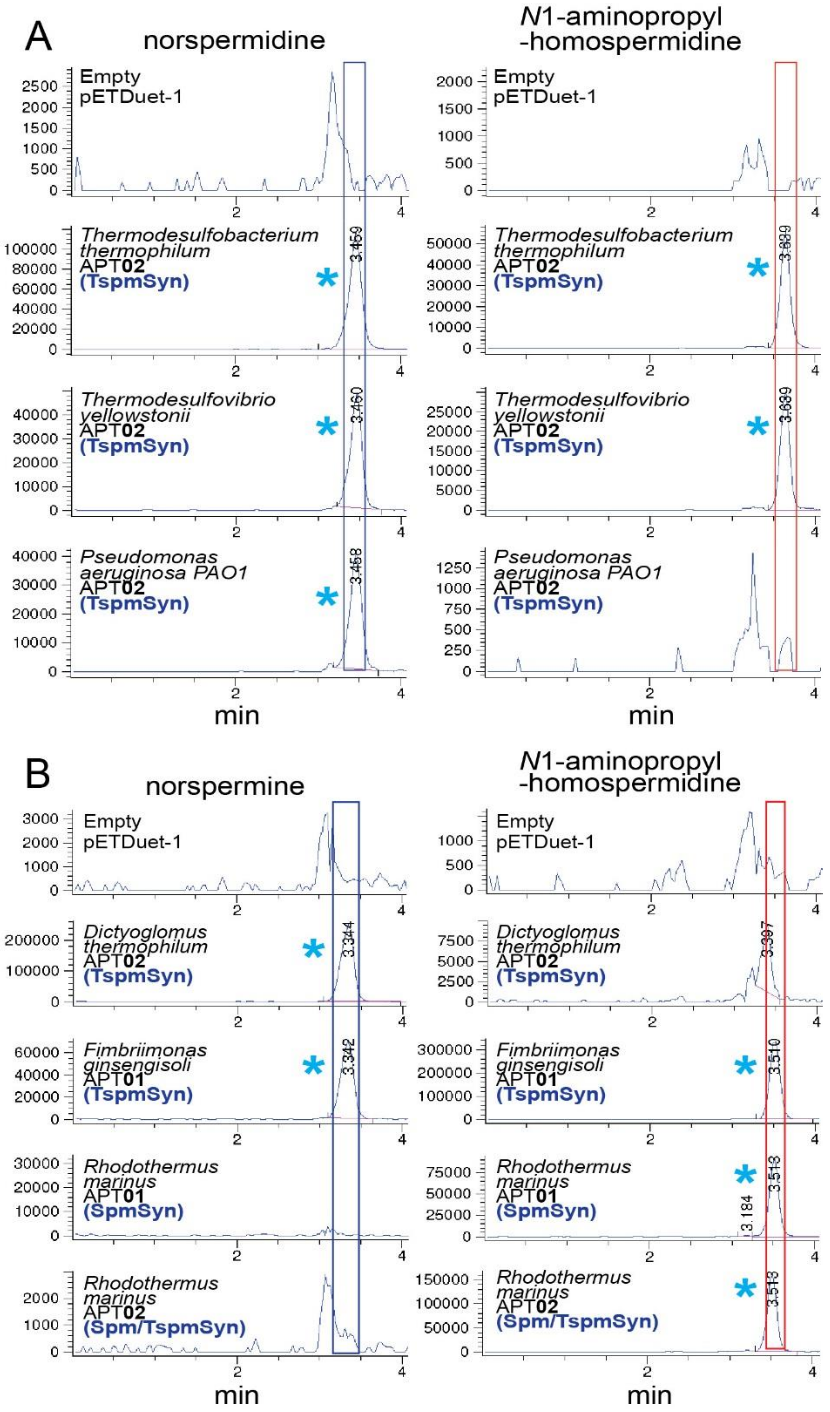

Figure S7. Expression of aminopropyltransferases in spermidine-devoid *E. coli* BL21*speE* grown with 0.5 mM norspermidine or homospermidine. *A* and *B* are independent experiments. Left panel samples were grown with 0.5 mM norspermidine, right panel with 0.5 mM homospermidine in M9 medium. Within *A* or *B*, cultures were grown and processed in parallel. Shown are the LC-MS Extracted Ion Chromatograms for tetrabenzoylated norspermine (Nspd, EIC = 605:606, blue box) and tetrabenzoylated N^1-aminopropylhomospermidine (N^1-APHspd, EIC = 619.02:620.02, red box). The presence of peaks for Nspd or N^1-APHspd are highlighted by blue asterisks. SpmSyn, spermine synthase; TspmSyn, thermospermine synthase. All genes were expressed from pETDuet-1.

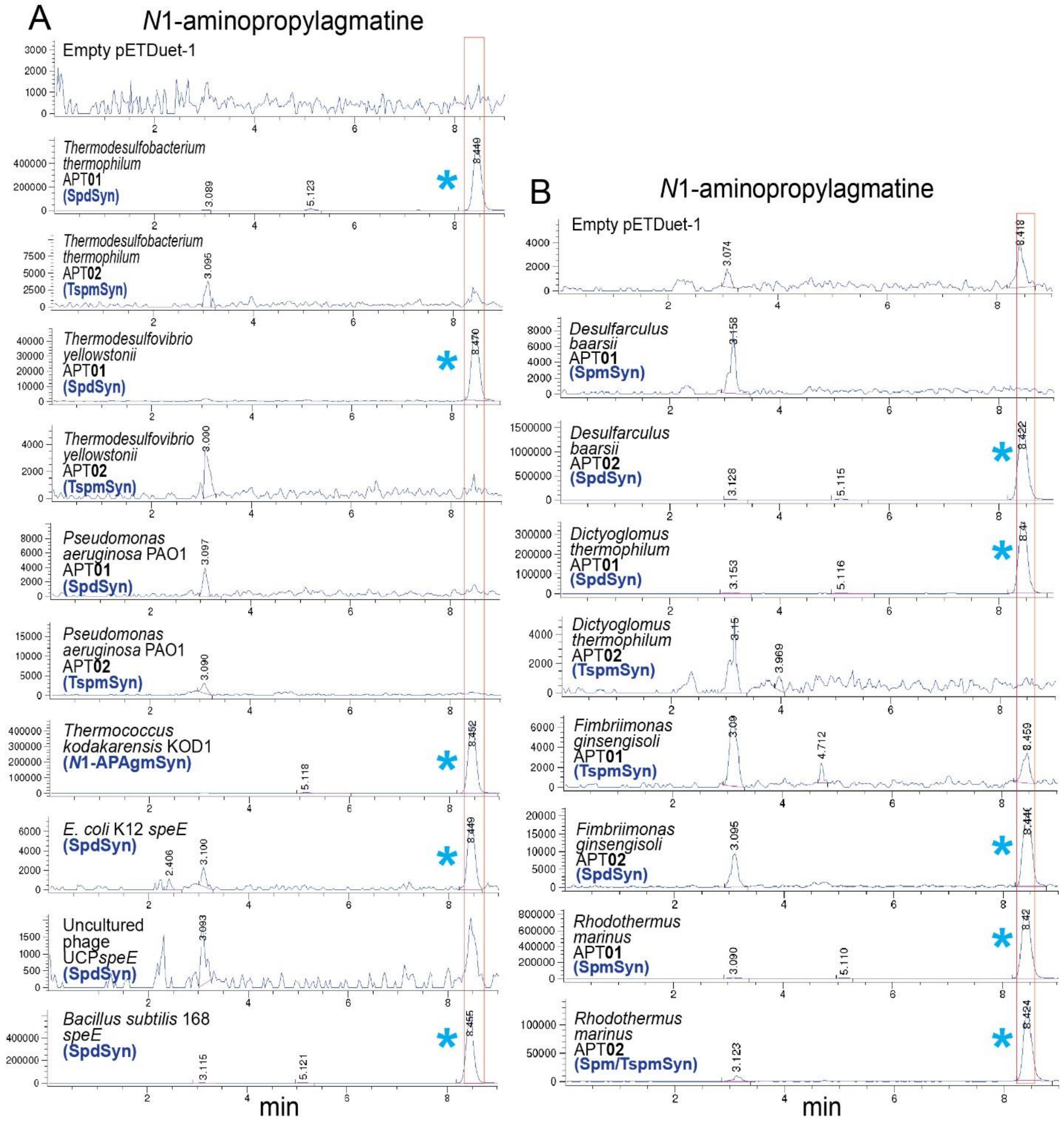

Figure S8. Expression of aminopropyltransferases in *E. coli* BL21*speB* grown with 0.3 mM L-arginine. *A* and *B* are independent experiments. Within *A* or *B*, cultures were grown and processed in parallel. Shown are the LC-MS Extracted Ion Chromatograms for tetrabenzoylated N^1-aminopropylated agmatine (N^1-APAgm, EIC = 603.98:604.98, red boxes). The presence of peaks for N^1-APAgm are highlighted by blue asterisks. SpmSyn, spermine synthase; TspmSyn, thermospermine synthase; SpdSyn, spermidine synthase; N^1-APAgmSyn, N^1-aminopropylagmatine synthase. All genes were expressed from pETDuet-1.

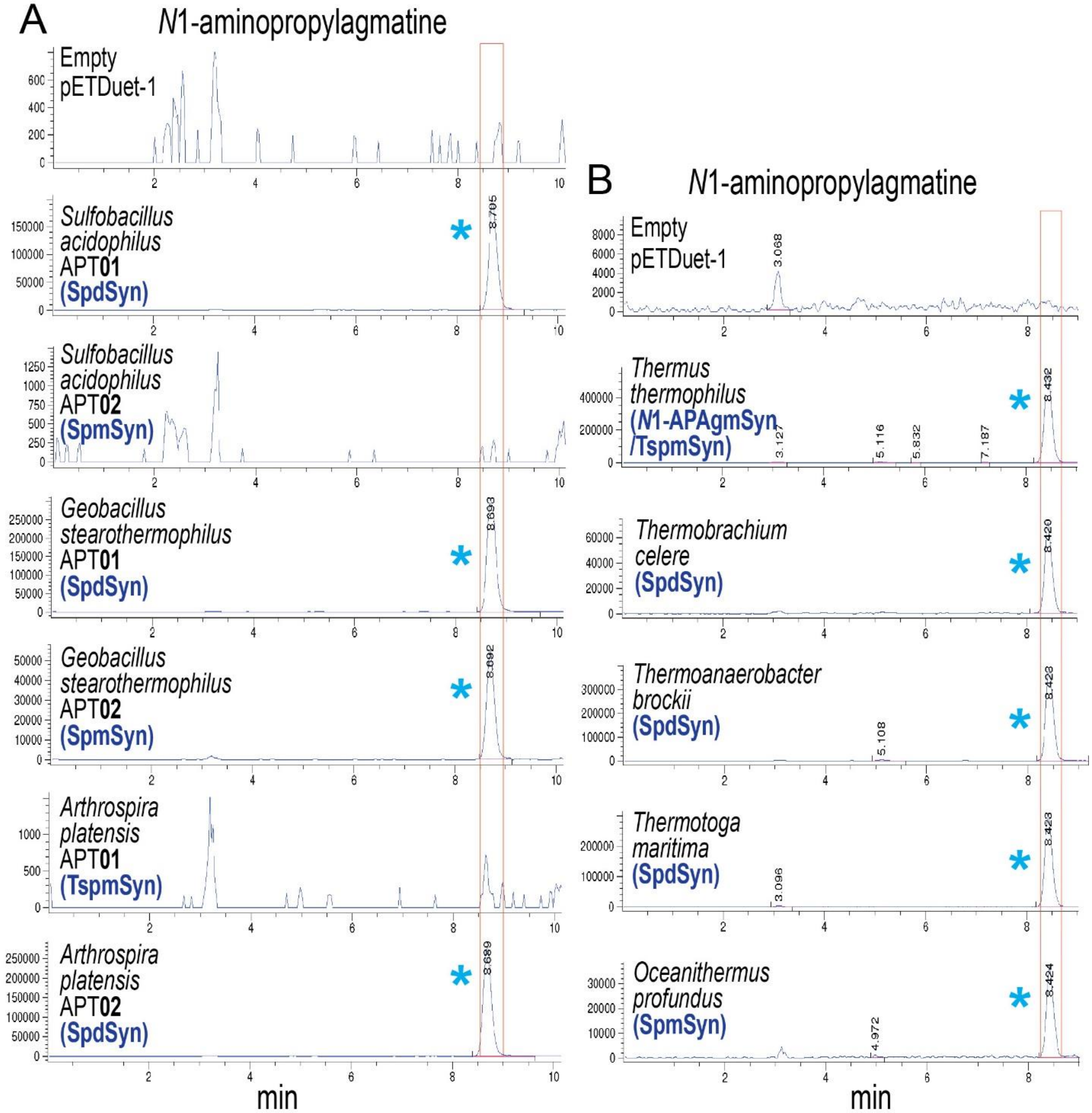

Figure S9. Expression of aminopropyltransferases in *E. coli* BL21*speB* grown with 0.3 mM L-arginine. *A* and *B* are independent experiments. Within *A* or *B*, cultures were grown and processed in parallel. Shown are the LC-MS Extracted Ion Chromatograms for tetrabenzoylated N^1-aminopropylated agmatine (N^1-APAgm, EIC = 603.98:604.98, red boxes). The presence of peaks for N^1-APAgm are highlighted by blue asterisks. SpmSyn, spermine synthase; TspmSyn, thermospermine synthase; SpdSyn, spermidine synthase; N^1-APAgmSyn, N^1-aminopropylagmatine synthase. All genes were expressed from pETDuet-1.

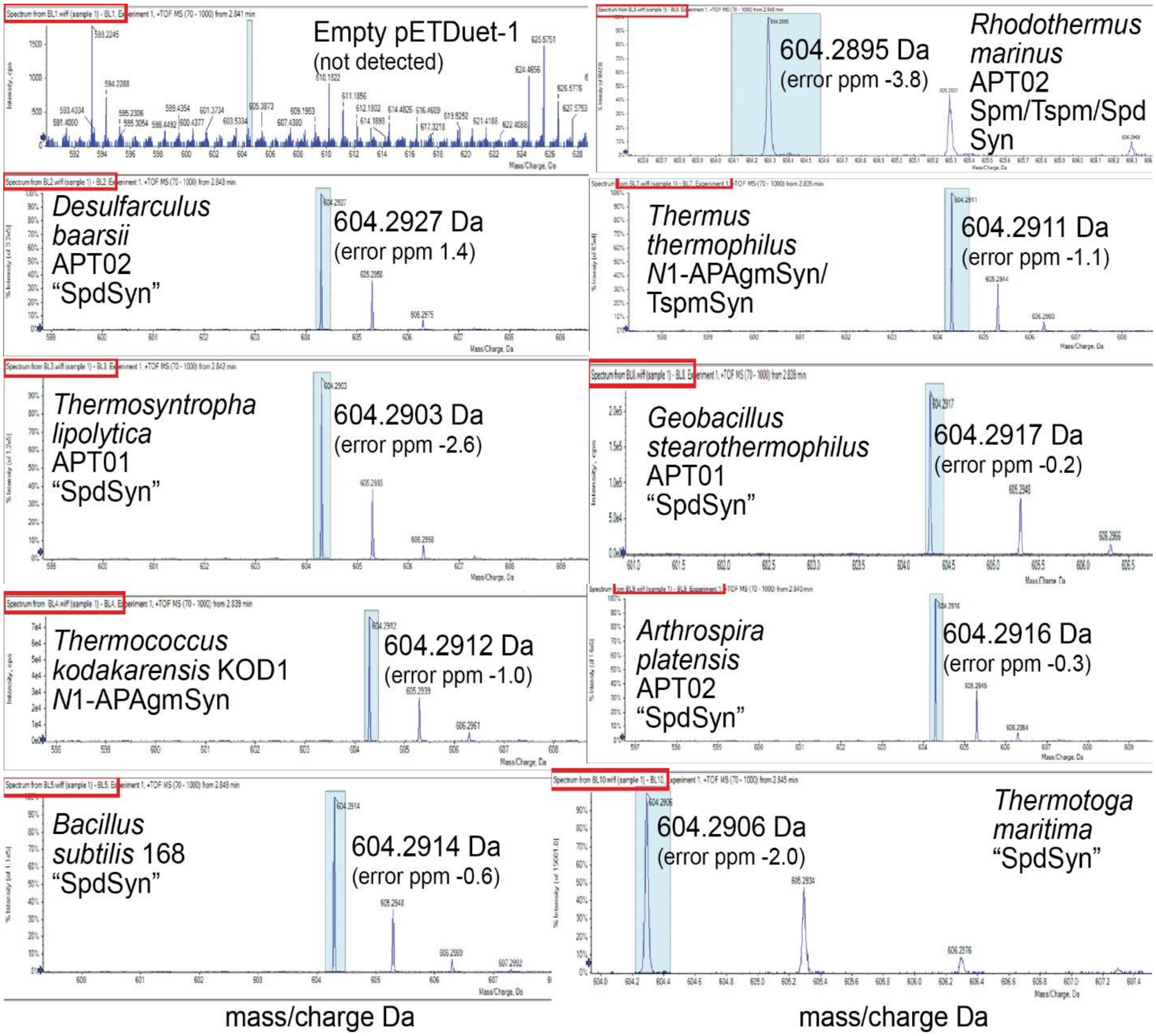

Figure S10. LC-HRMS detection in positive polarity mode of tetrabenzoylated *N*[1]-aminopropylagmatine after expression of aminopropyltransferases in *E. coli* BL21*speB* grown with 0.3 mM L-arginine. The theoretical mass for tetrabenzoylated *N*[1]-aminopropylagmatine is 604.29183, and the detected peak is highlighted in blue. SpmSyn, spermine synthase; TspmSyn, thermospermine synthase; SpdSyn, spermidine synthase; *N*[1]-APAgmSyn, *N*[1]-aminopropylagmatine synthase. All genes were expressed from pETDuet-1.

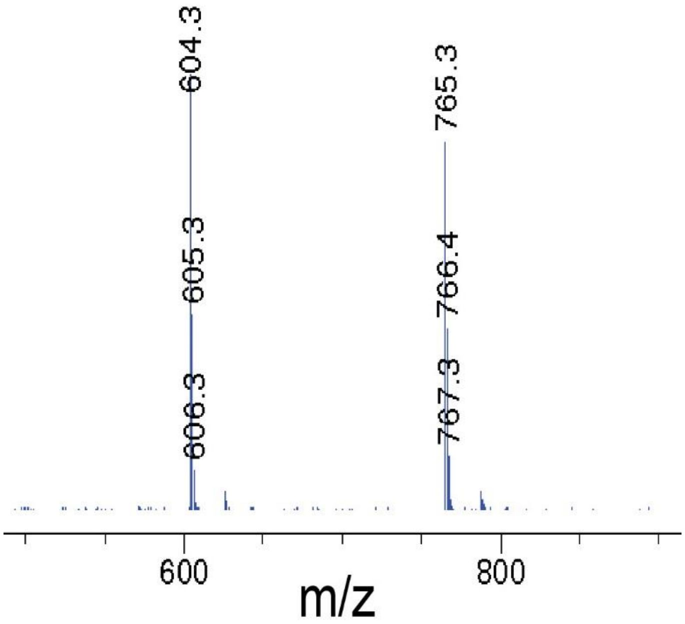

Figure S11. LC-MS mass spectrum of a peak corresponding to the Extracted Ion Chromatogram of tetrabenzoylated *N*¹-aminopropylagmatine and pentabenzoylated *N*¹²-guanidinothermospermine. Polyamines were extracted from *E. coli* BL21*speB* grown with 3 mM L-arginine and co-expressing the *Dictyoglomus thermophilum* spermidine synthase/*N*¹-aminopropylagmatine synthase (SpdSyn/ *N*¹-APAgmSyn) from pETDuet-1 and thermospermine synthase (TspmSyn) from pACYCDuet-1 (Figure 8, main manuscript). The peak corresponding to the EICs for tetrabenzoylated *N*¹-aminopropylagmatine and pentabenzoylated *N*¹²-guanidinothermospermine eluted at very similar times. Shown is the mass spectrum for the peak eluting at 8.851 min of the EIC = 765.07:766.07 for pentabenzoylated *N*¹²-guanidinothermospermine, which also contains the mass of tetrabenzoylated *N*¹-aminopropylagmatine (m/z = 604.3).
